# Supplementary material for: Identification and Characterization of α-Glucosidase Inhibition Flavonol Glycosides from Jack Bean (Canavalia ensiformis (L.) DC
Source: Molecules. 2020 May 27;25(11):2481. doi: 10.3390/molecules25112481 (PMC7321285; doi:10.3390/molecules25112481)
Supplement: Supplementary file 1 [file molecules-25-02481-s001.pdf]

SUPPLEMENT INFORMATION

Identification and characterization of α-glucosidase inhibition flavonol glycosides from jack bean (*Canavalia ensiformis* (L.) DC.

Anita M. Sutedja[1,2,3], Emiko Yanase[1,*], Irmanida Batubara[4,5], Dedi Fardiaz[2] and Hanifah N. Lioe[2]

1 Faculty of Applied Biological Sciences, Graduate School of Applied Biological Sciences, Gifu University, 1-1 Yanagido, Gifu City, Gifu 501-1193, Japan; e-yanase@gifu-u.ac.jp
2 Department of Food Science and Technology, Faculty of Agricultural Engineering and Technology, IPB University, Kampus IPB Dramaga, Bogor 16680, West Java, Indonesia; hanifahlioe@apps.ipb.ac.id; dedi_fardiaz@yahoo.com.sg
3 Department of Food Technology, Faculty of Agricultural Technology, Widya Mandala Catholic University Surabaya, Jalan Dinoyo 42-44, Surabaya 60265, East Java, Indonesia; maya@ukwms.ac.id
4 Department of Chemistry, Faculty of Mathematics and Natural Science, IPB University, Kampus IPB Dramaga, Bogor 16680, West Java, Indonesia; imebatubara@gmail.com
5 Tropical Biopharmaca Research Center IPB University, Kampus IPB Taman Kencana No 3, Bogor 16128, West Java, Indonesia
* Correspondence: e-yanase@gifu-u.ac.jp; Tel.: +81-58 293 2914

Table of Content

Figure S1. ^{1}H NMR spectrum of compound **1** (CD_3OD, 800 MHz)	4
Figure S2. ^{13}C NMR spectrum of compound **1** (CD_3OD, 800 MHz)	5
Figure S3. ^{1}H-^{1}H COSY of compound **1** (CD_3OD, 800 MHz)	6
Figure S4. HMQC of compound **1** (CD_3OD, 800 MHz)	7
Figure S5. HMBC of compound **1** (CD_3OD, 800 MHz)	8
Figure S6. ^{1}H NMR spectrum of compound **2** (CD_3OD, 800 MHz)	9
Figure S7. ^{13}C NMR spectrum of compound **2** (CD_3OD, 800 MHz)	10
Figure S8. ^{1}H-^{1}H COSY of compound **2** (CD_3OD, 800 MHz)	11
Figure S9. HMQC of compound **2** (CD_3OD, 800 MHz)	12
Figure S10. HMBC of compound **2** (CD_3OD, 800 MHz)	13
Figure S11. ^{1}H NMR spectrum of compound **3** (CD_3OD, 800 MHz)	14
Figure S12. ^{13}C NMR spectrum of compound **3** (CD_3OD, 800 MHz)	15
Figure S13. ^{1}H-^{1}H COSY of compound **3** (CD_3OD, 800 MHz)	16
Figure S14. HMQC of compound **3** (CD_3OD, 800 MHz)	17
Figure S15. HMBC of compound **3** (CD_3OD, 800 MHz)	18
Figure S16. ^{1}H NMR spectrum of compound **4** (CD_3OD, 600 MHz)	19
Figure S17. ^{13}C NMR spectrum of compound **4** (CD_3OD, 600 MHz)	20
Figure S18. ^{1}H-^{1}H COSY of compound **4** (CD_3OD, 600 MHz)	21
Figure S19. HMQC of compound **4** (CD_3OD, 600 MHz)	22
Figure S20. HMBC of compound **4** (CD_3OD, 600 MHz)	23
Figure S21. LC/ESI/MS/MS data of compound **1**	24
Figure S22. LC/ESI/MS/MS data of compound **2**	25
Figure S23. LC/ESI/MS/MS data of compound **3**	26
Figure S24. LC/ESI/MS/MS data of compound **4**	27
Figure S25. LC/ESI/MS/MS data of compound **5**	28
Figure S26. LC/ESI/MS/MS data of compound **6**	29
Figure S27. LC/ESI/MS/MS data of compound **7**	30
Figure S28. LC/ESI/MS/MS data of compound **8**	31
Figure S29. LC/ESI/MS/MS data of compound **9**	32
Figure S30. LC/ESI/MS/MS data of compound **10**	33
Figure S31. LC/ESI/MS/MS data of compound **11**	34
Figure S32. LC/ESI/MS/MS data of compound **12**	35
Figure S33. LC/ESI/MS/MS data of compound **13**	36
Figure S34. LC/ESI/MS/MS data of compound **14**	37
Figure S35. GC-MS total ion chromatogram and extracted ion chromatogram of trimethylsilyl (TMS) derivatives of galactose	38

Figure S36. GC-MS total ion chromatogram and extracted ion chromatogram of trimethylsilyl (TMS) derivatives of glucose 39
Figure S37. GC-MS total ion chromatogram and extracted ion chromatogram of trimethylsilyl (TMS) derivatives of rhamnose 40
Figure S38. GC-MS total ion chromatogram and extracted ion chromatogram trimethylsilyl (TMS) derivatives of compound **1** 41
Figure S39. GC-MS total ion chromatogram and extracted ion chromatogram trimethylsilyl (TMS) derivatives of compound **2** 42
Figure S40. GC-MS total ion chromatogram and extracted ion chromatogram trimethylsilyl (TMS) derivatives of compound **3** 43
Figure S41. GC-MS total ion chromatogram and extracted ion chromatogram trimethylsilyl (TMS) derivatives of compound **4** 44
Figure S42. Chromatogram of compounds **1** (a) and **2** (b) 45
Figure S43. Chromatogram of compounds **3** (a) and **4** (b) 46

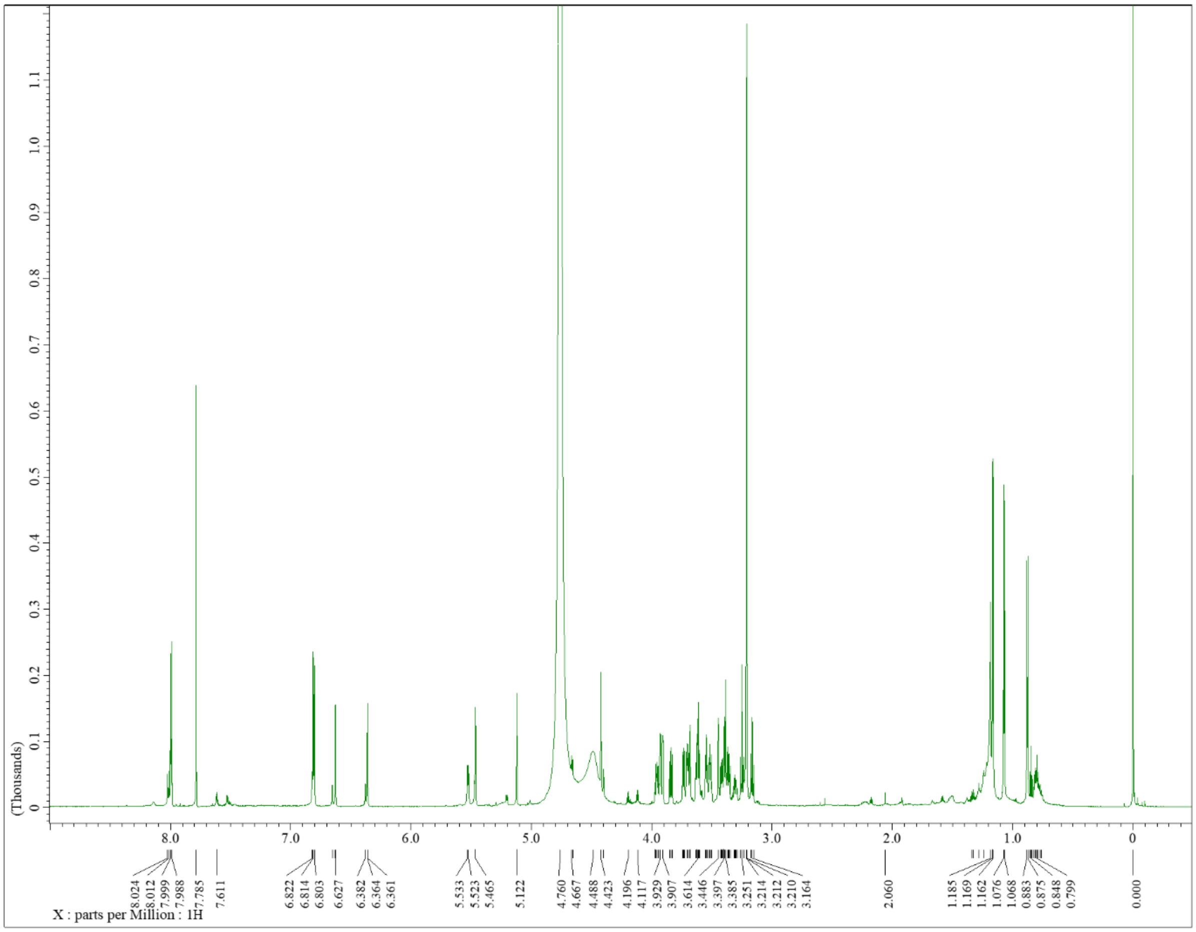

Figure S1. ^{1}H NMR spectrum of compound **1** (CD_3OD, 800 MHz)

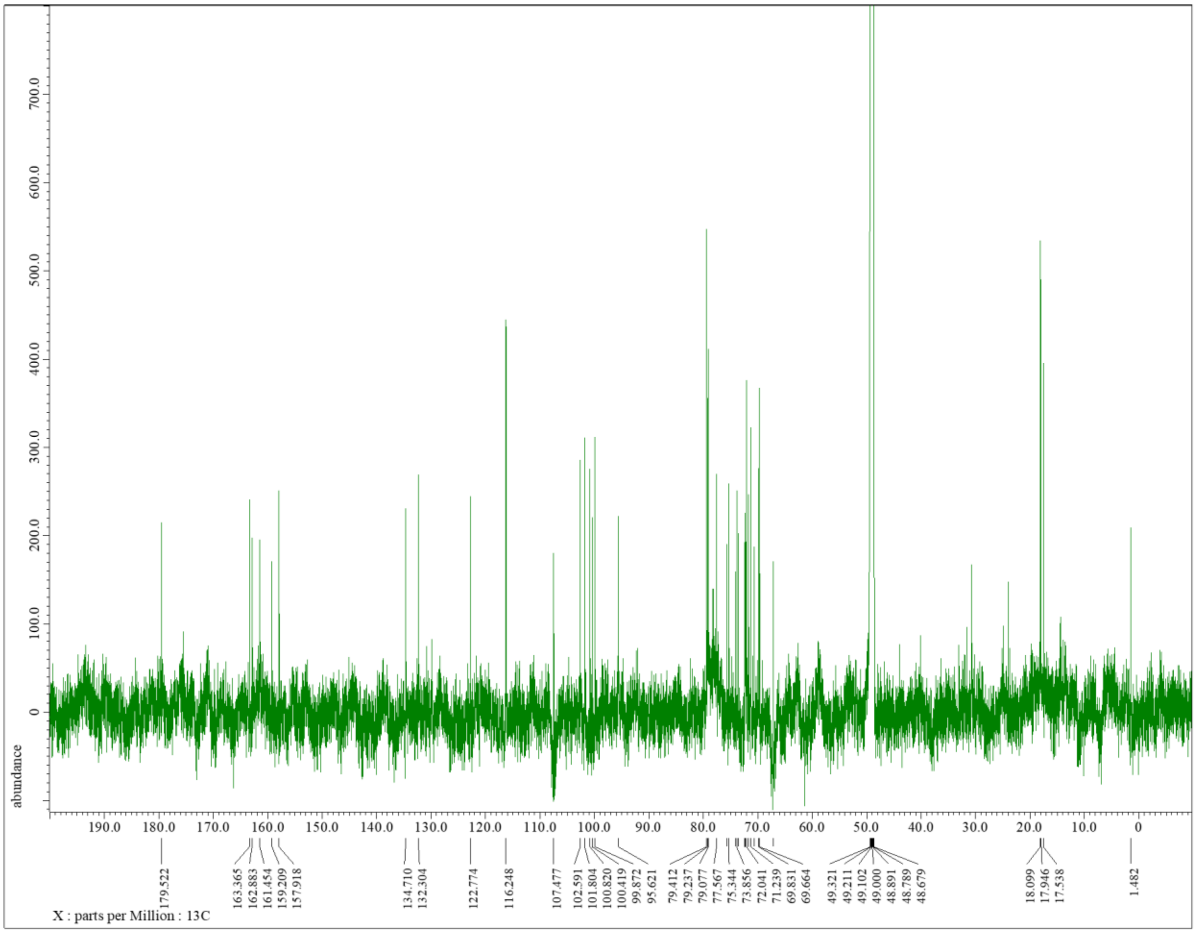

Figure S2. ^{13}C NMR spectrum of compound **1** (CD_3OD, 800 MHz)

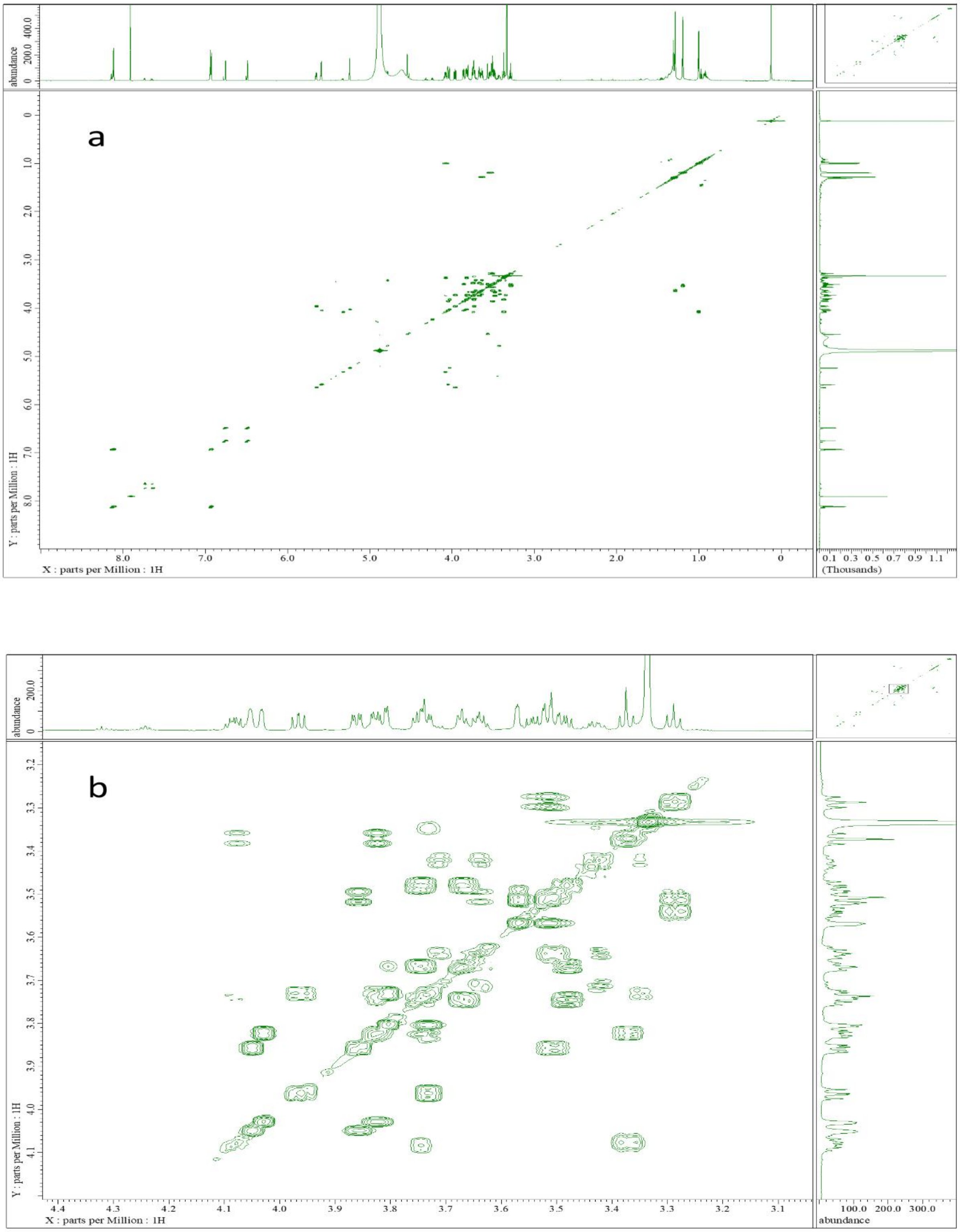

Figure S3. ${}^{1}H$-${}^{1}H$ COSY of compound **1** (CD_3OD, 800 MHz)

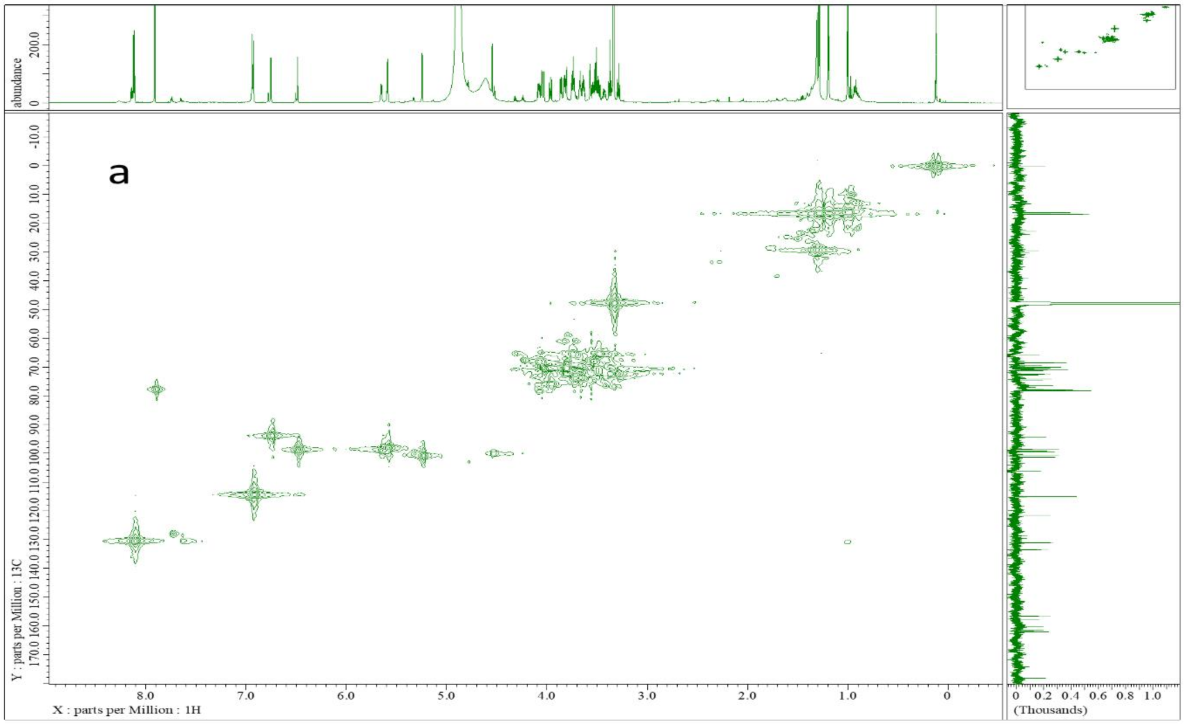

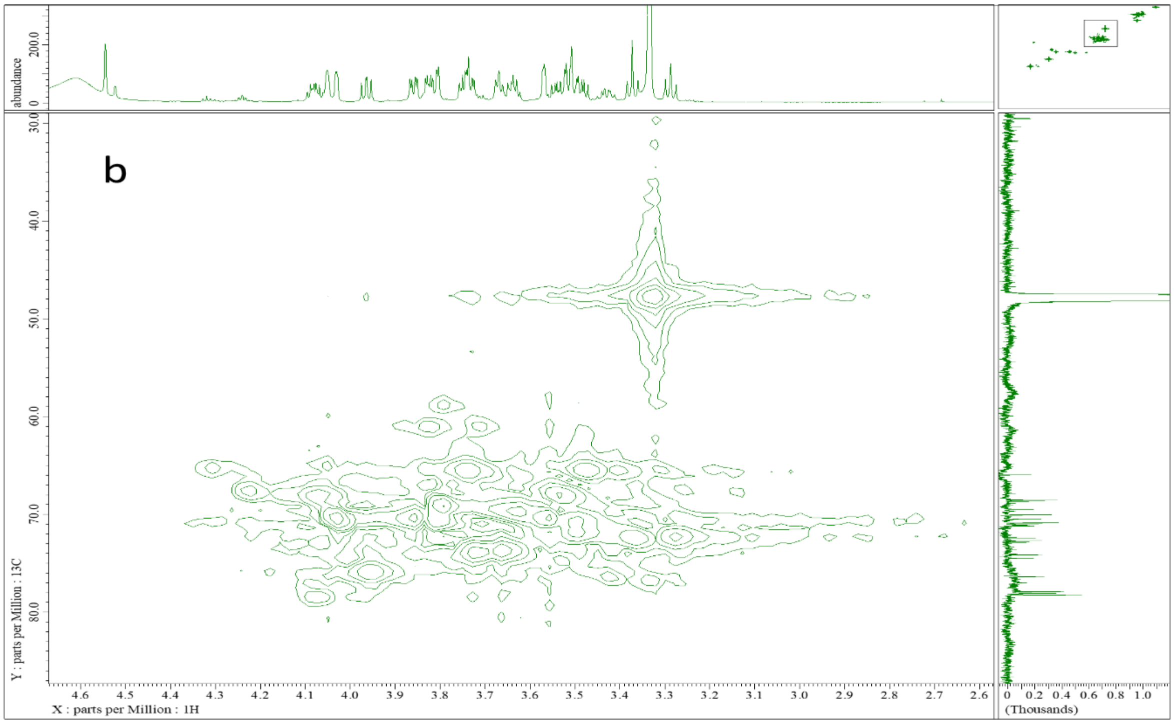

Figure S4. HMQC of compound **1** (CD_3OD, 800 MHz)

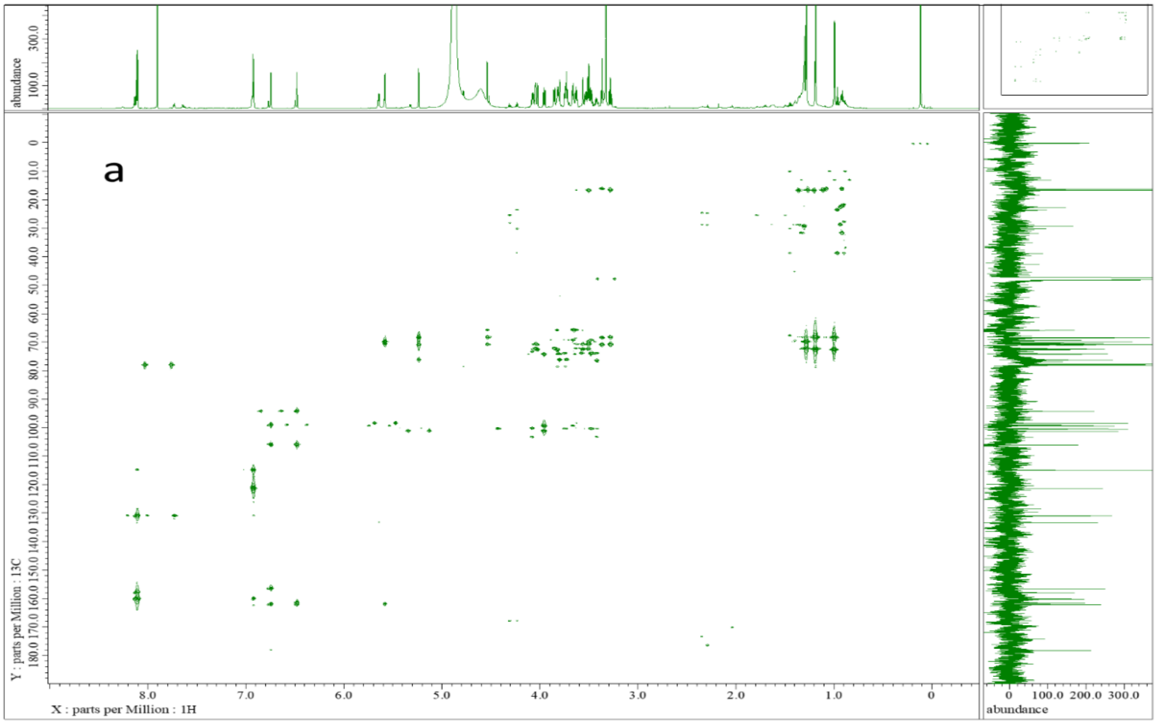

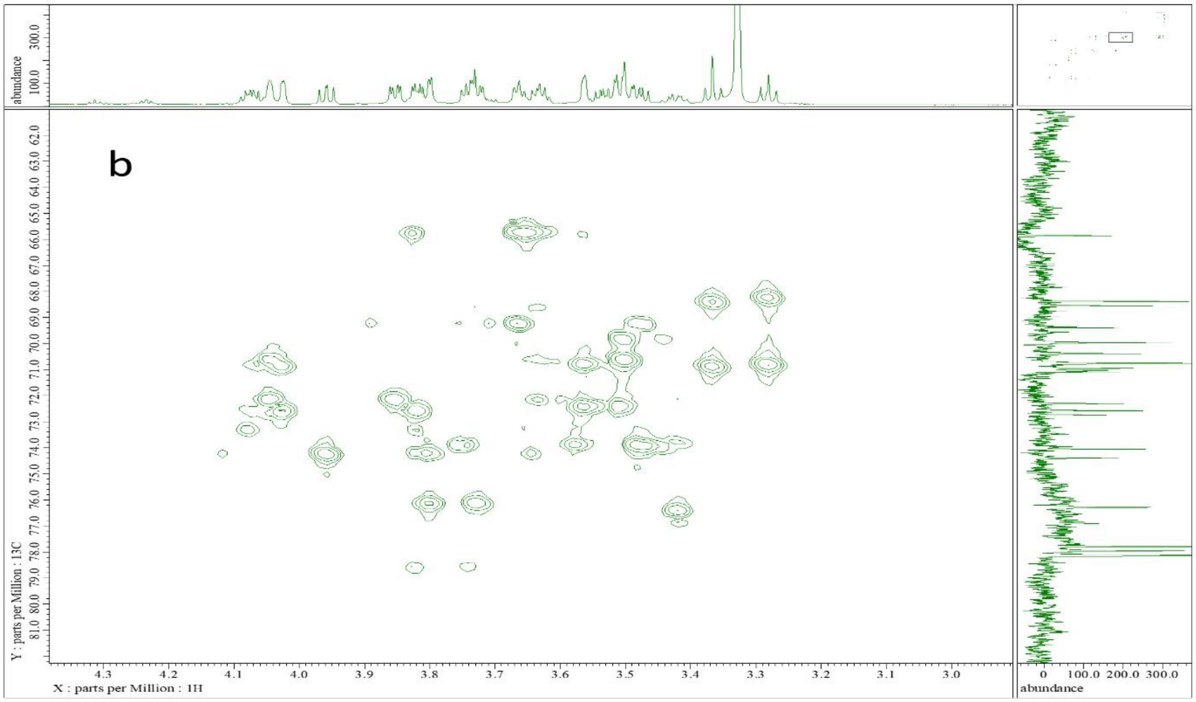

Figure S5. HMBC of compound **1** (CD_3OD, 800 MHz)

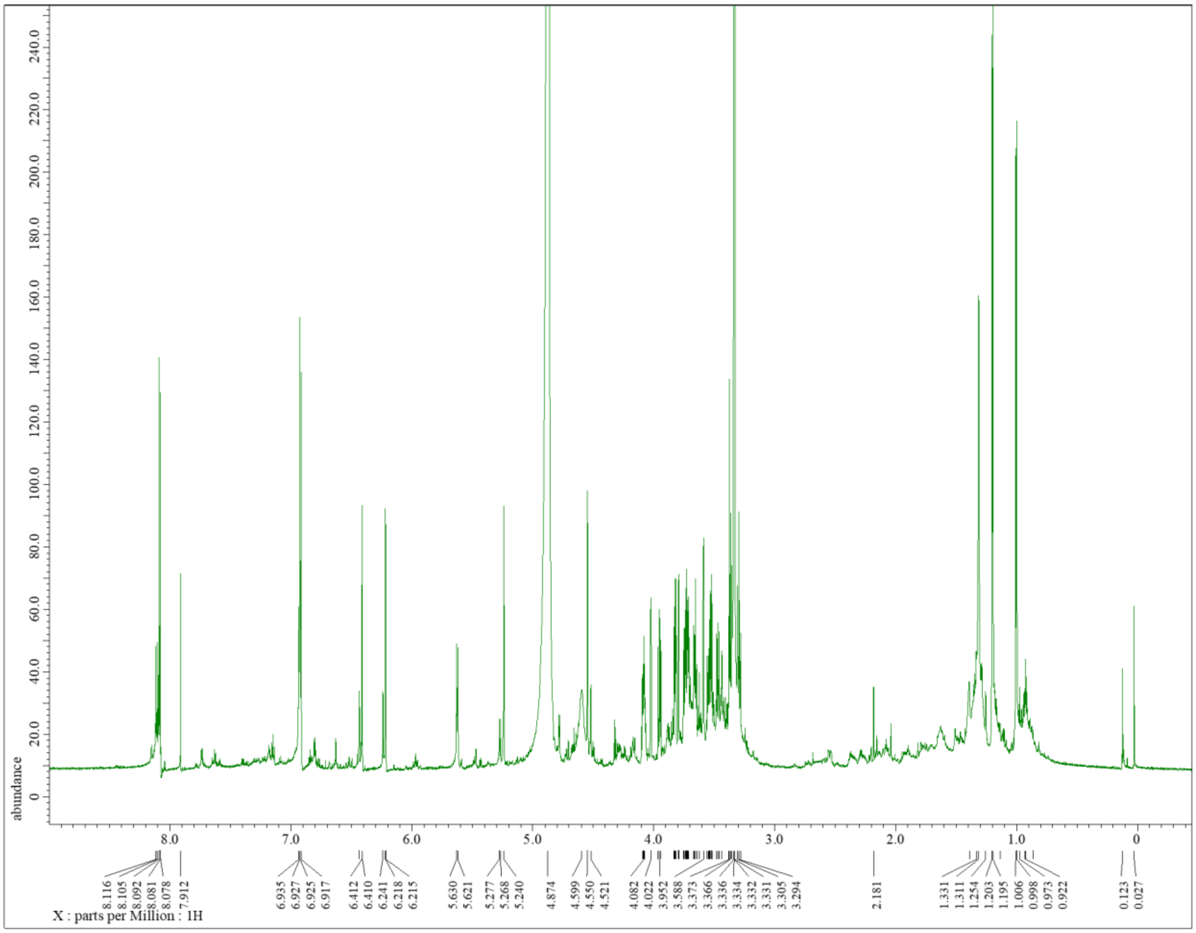

Figure S6. ^{1}H NMR spectrum of compound **2** (CD_3OD, 800 MHz)

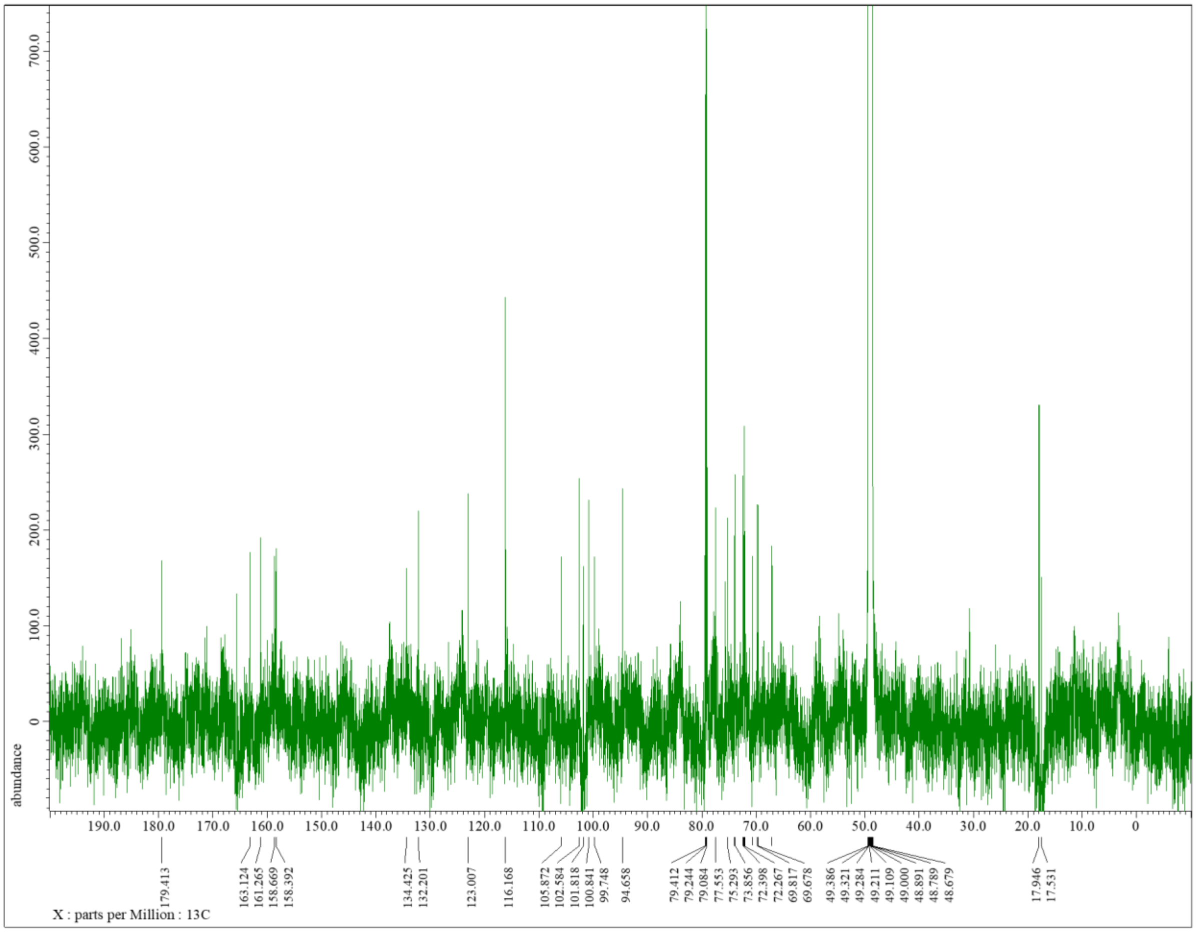

Figure S7. ^{13}C NMR spectrum of compound **2** (CD_3OD, 800 MHz)

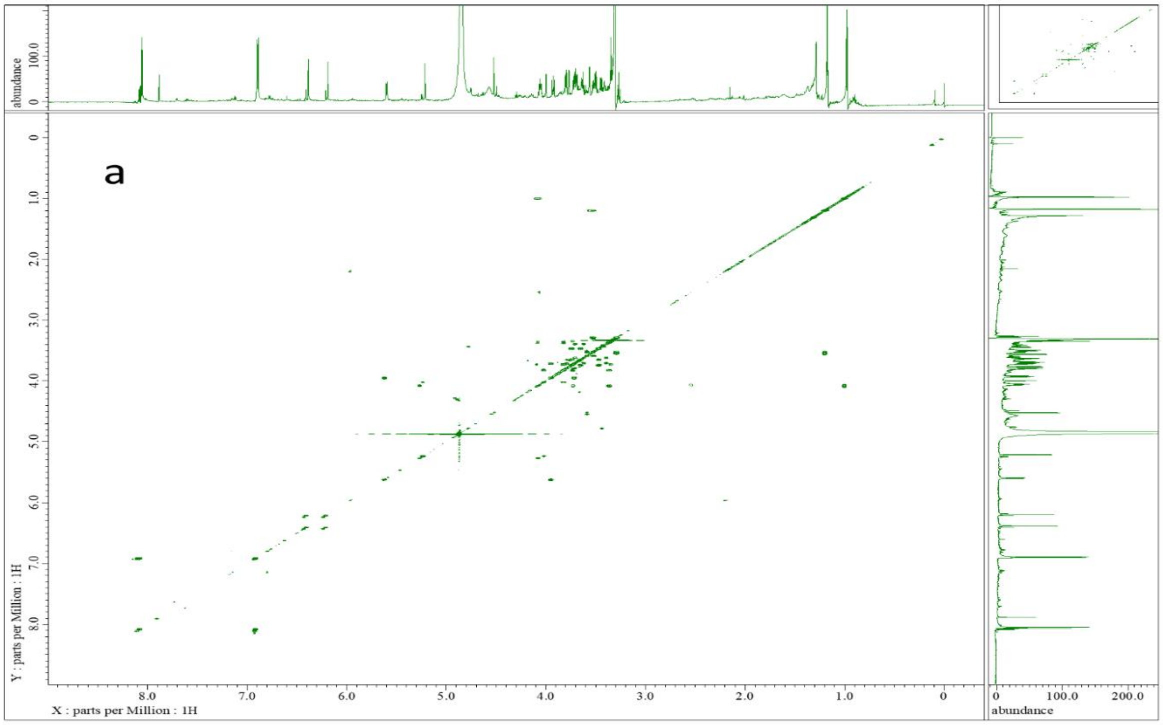

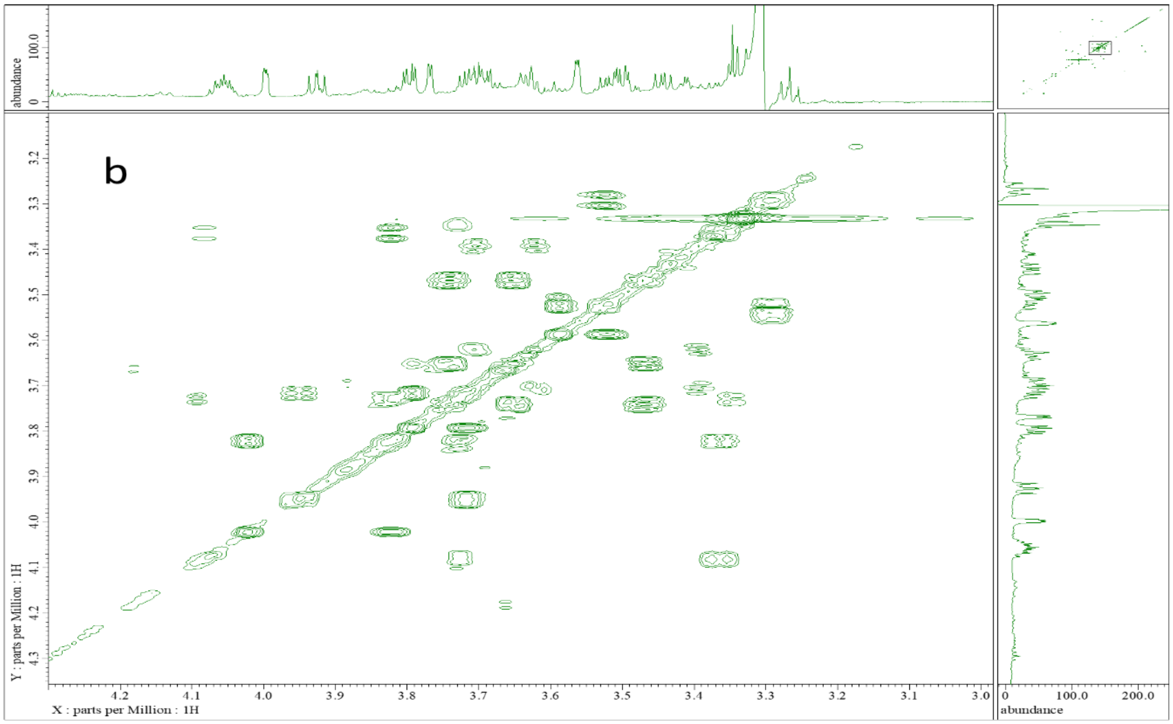

Figure S8. ^1H-^1H COSY of compound **2** (CD_3OD, 800 MHz)

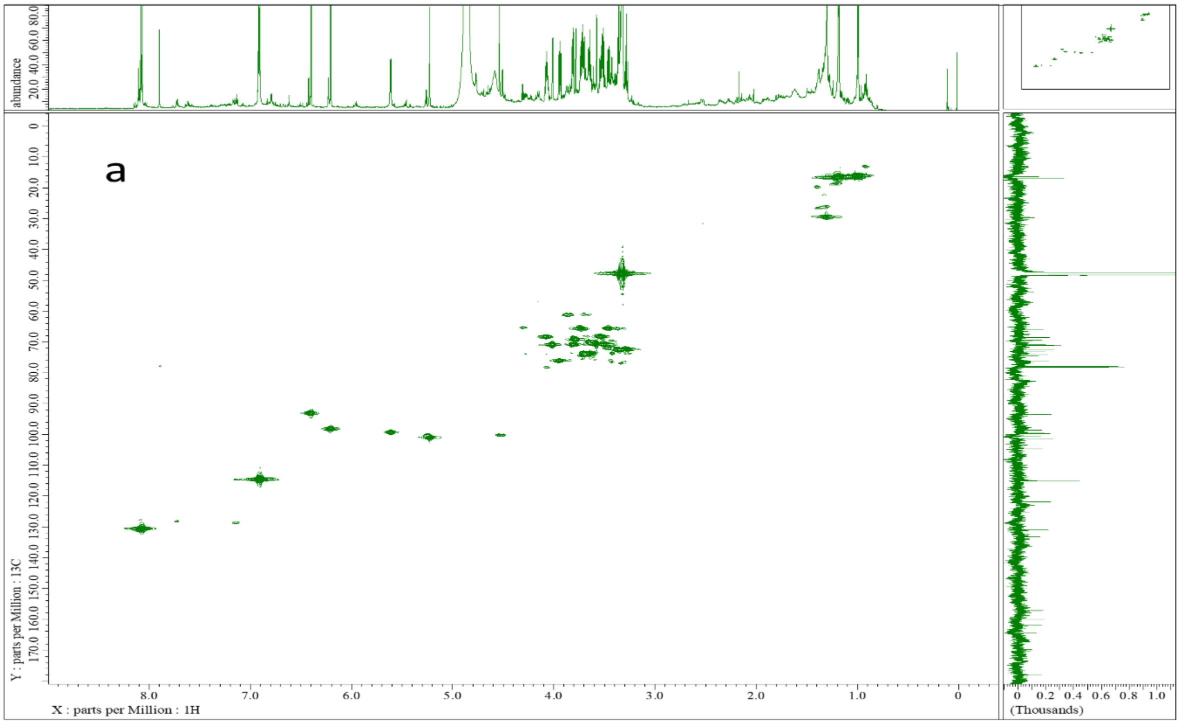

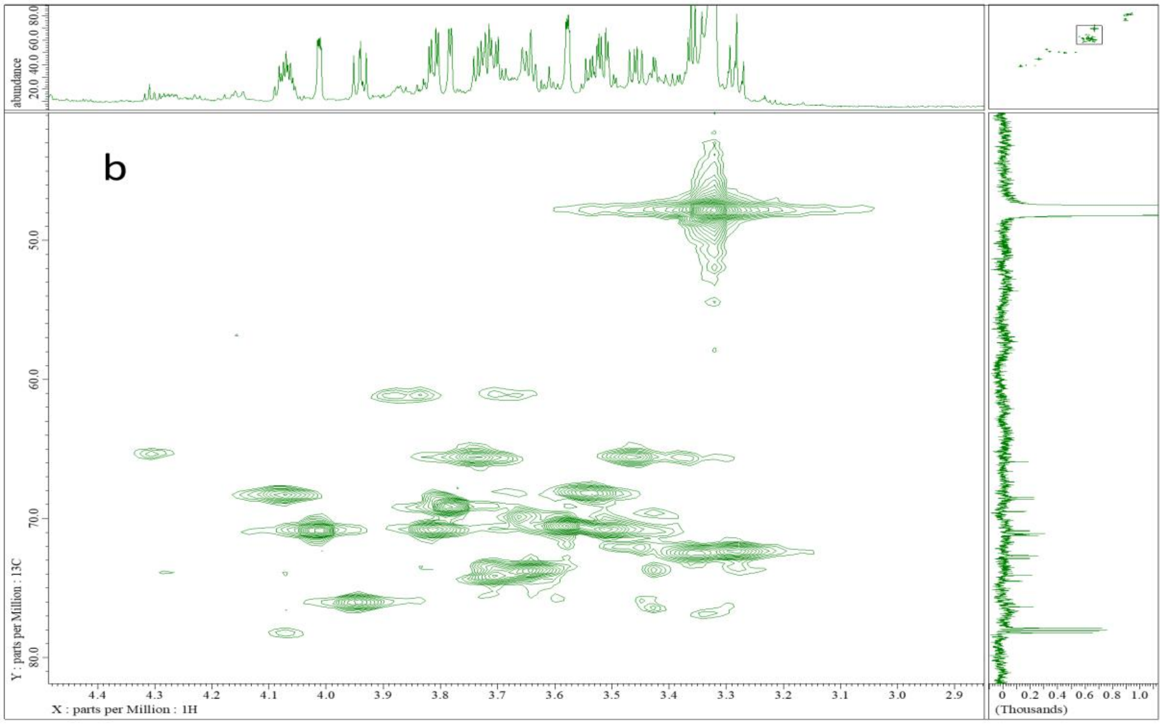

Figure S9. HMQC of compound **2** (CD_3OD, 800 MHz)

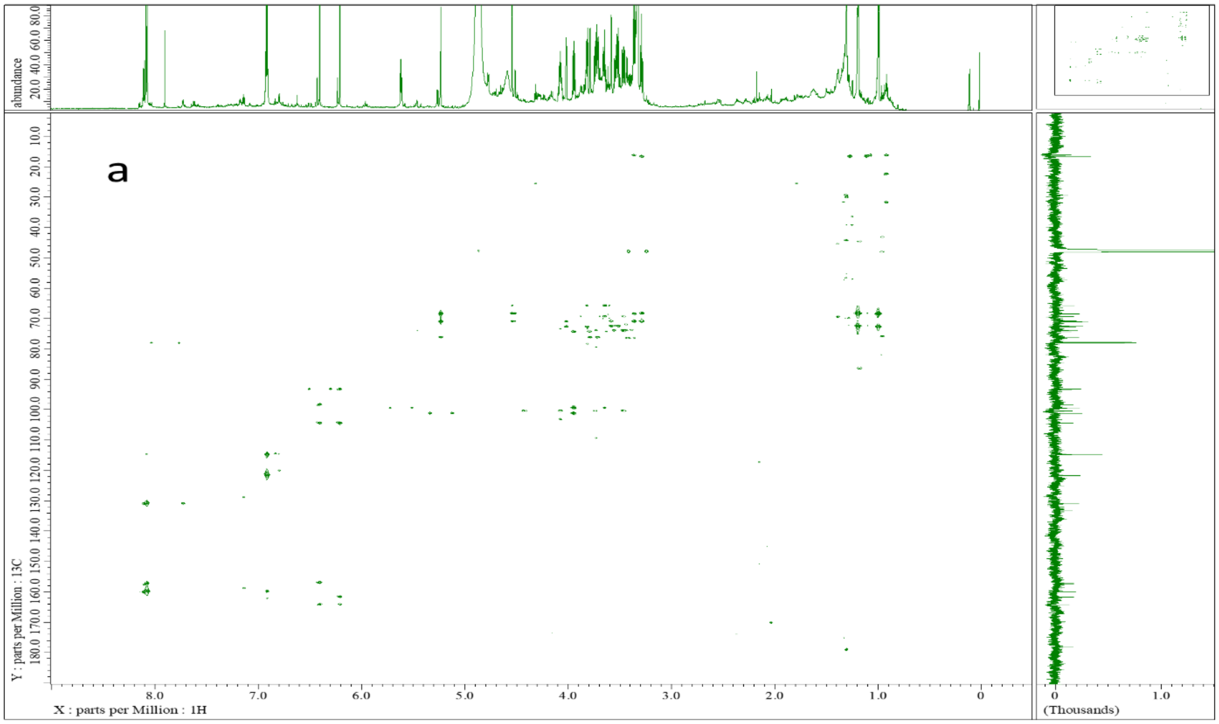

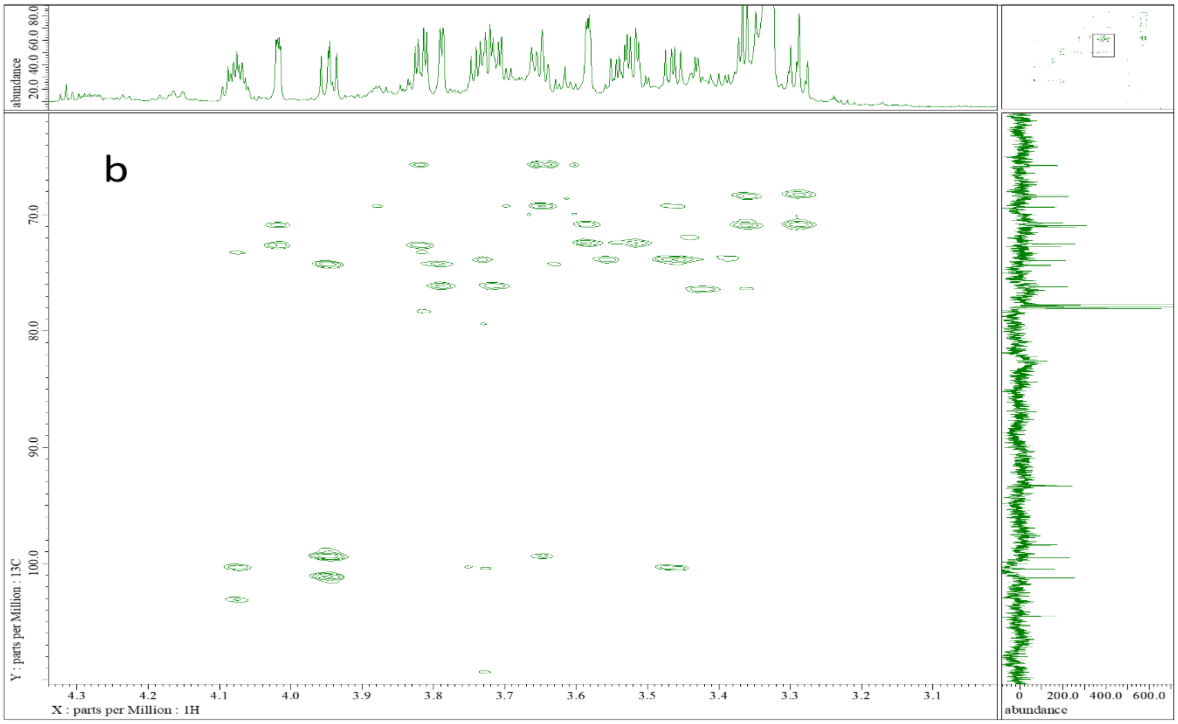

Figure S10. HMBC of compound **2** (CD_3OD, 800 MHz)

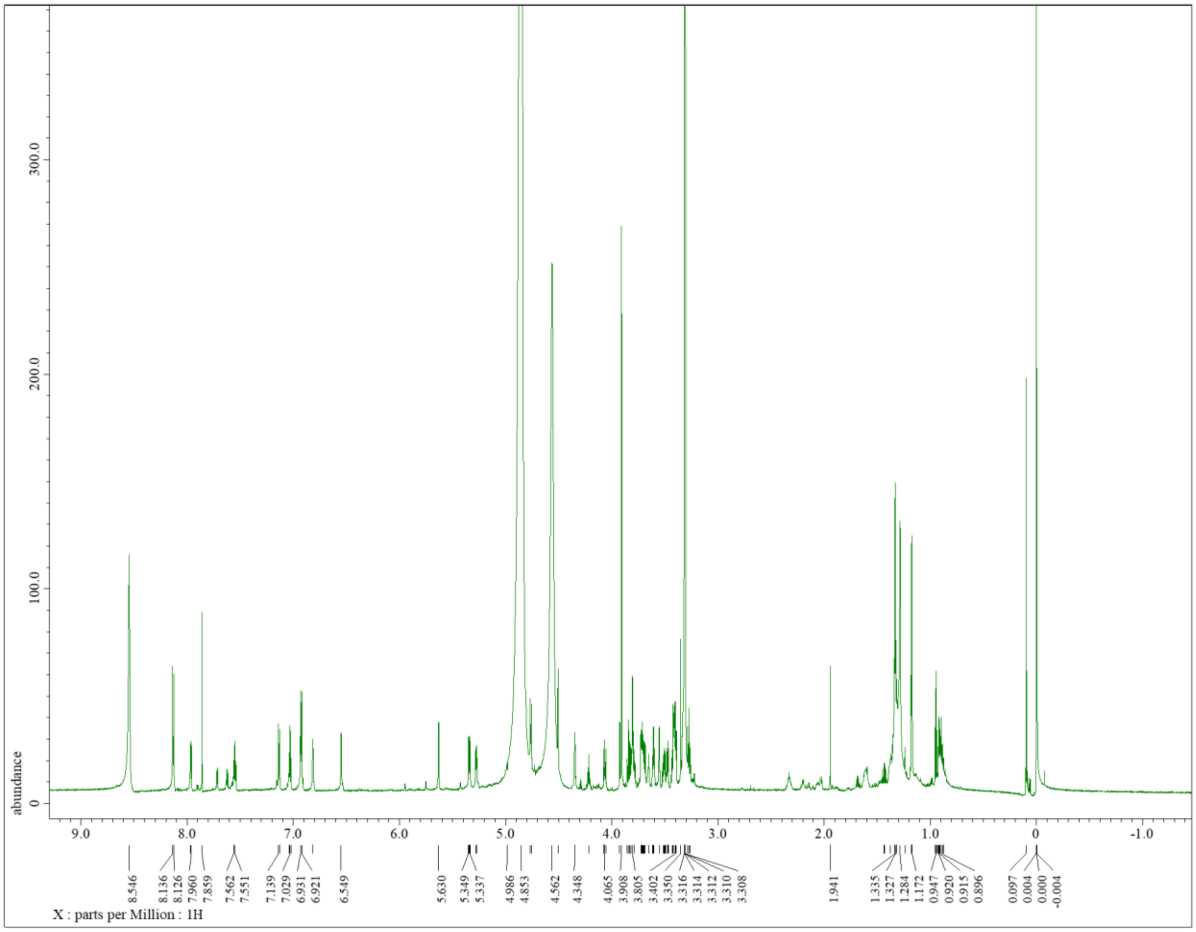

Figure S11. ^{1}H NMR spectrum of compound **3** (CD_3OD, 800 MHz)

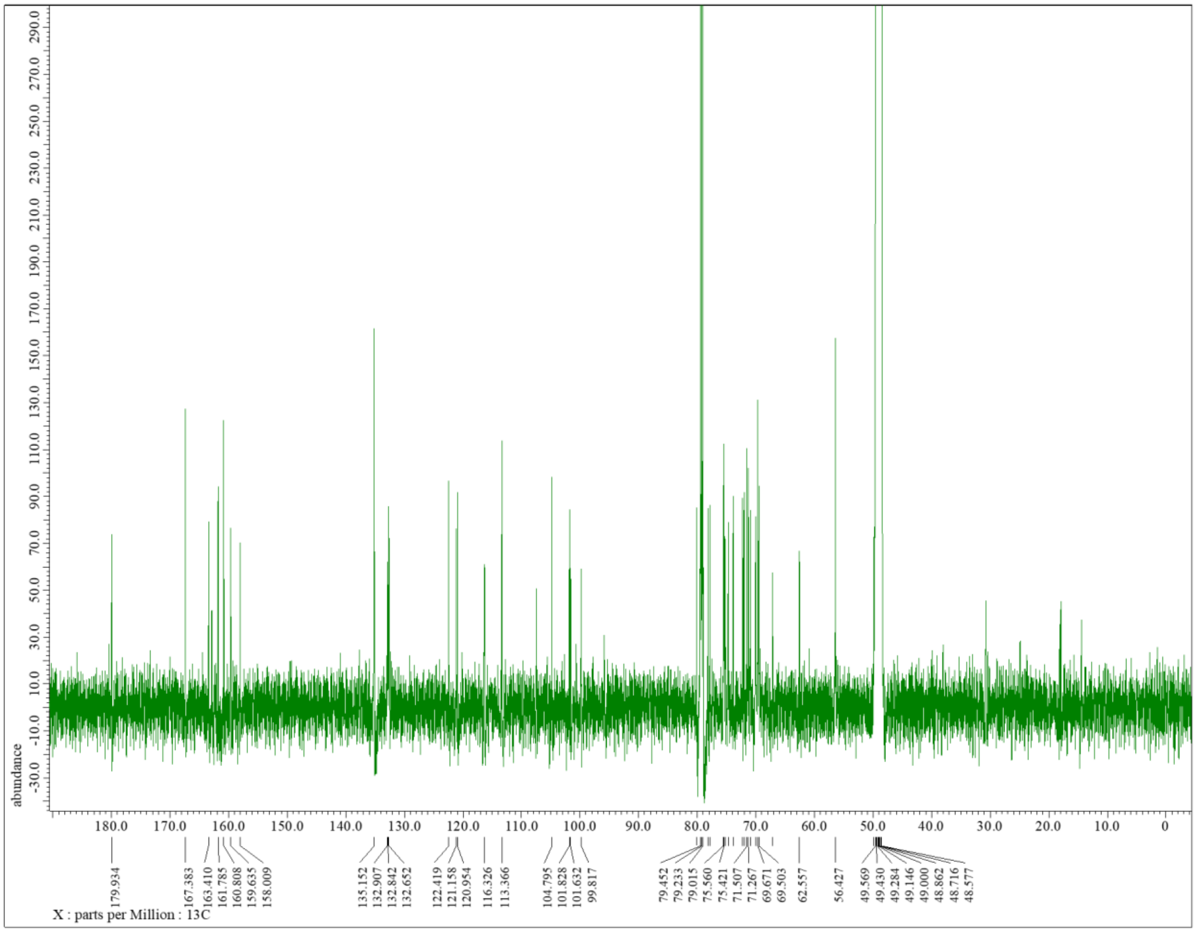

Figure S12. ^{13}C NMR spectrum of compound **3** (CD_3OD, 800 MHz)

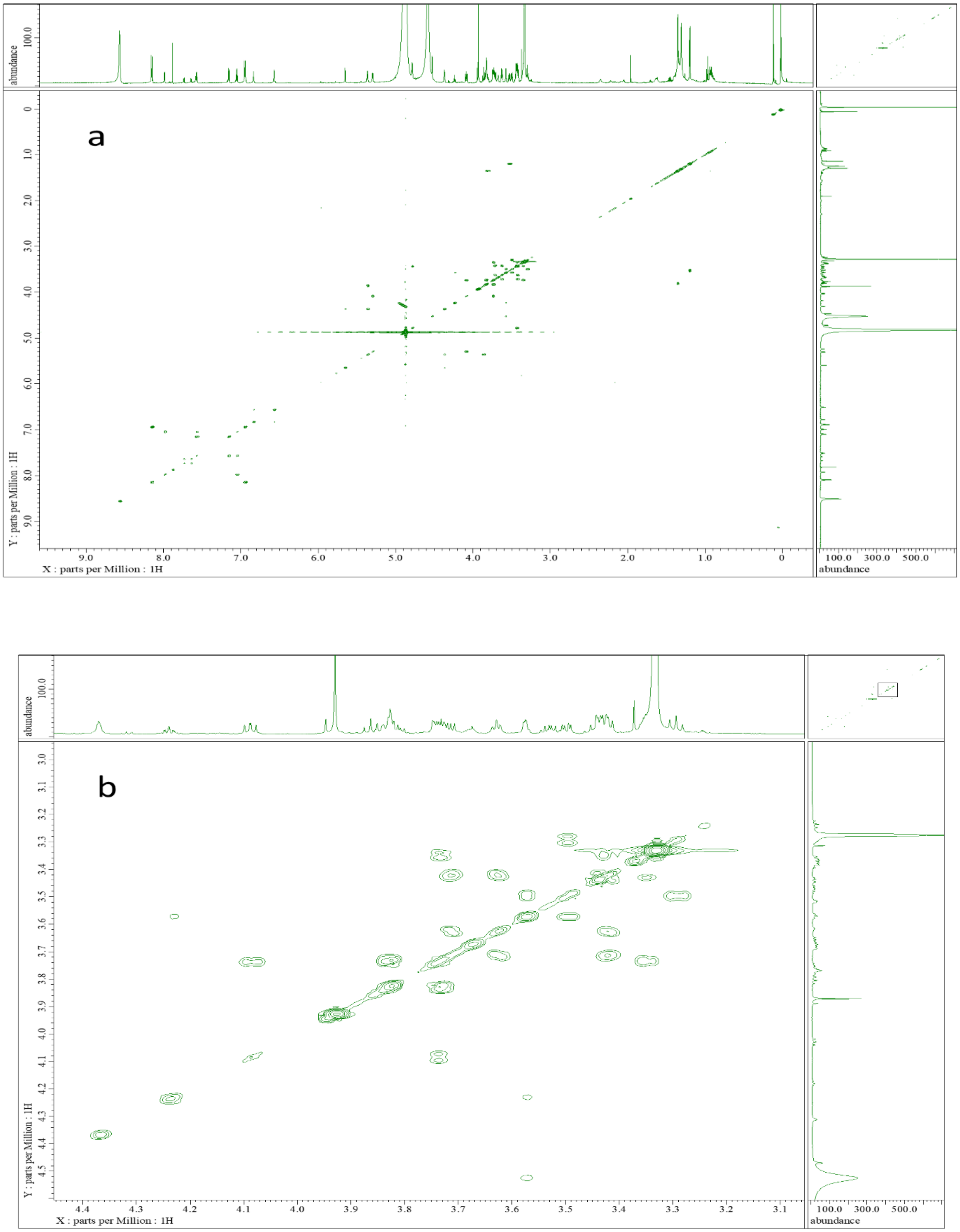

Figure S13. ^1H-^1H COSY of compound **3** (CD_3OD, 800 MHz)

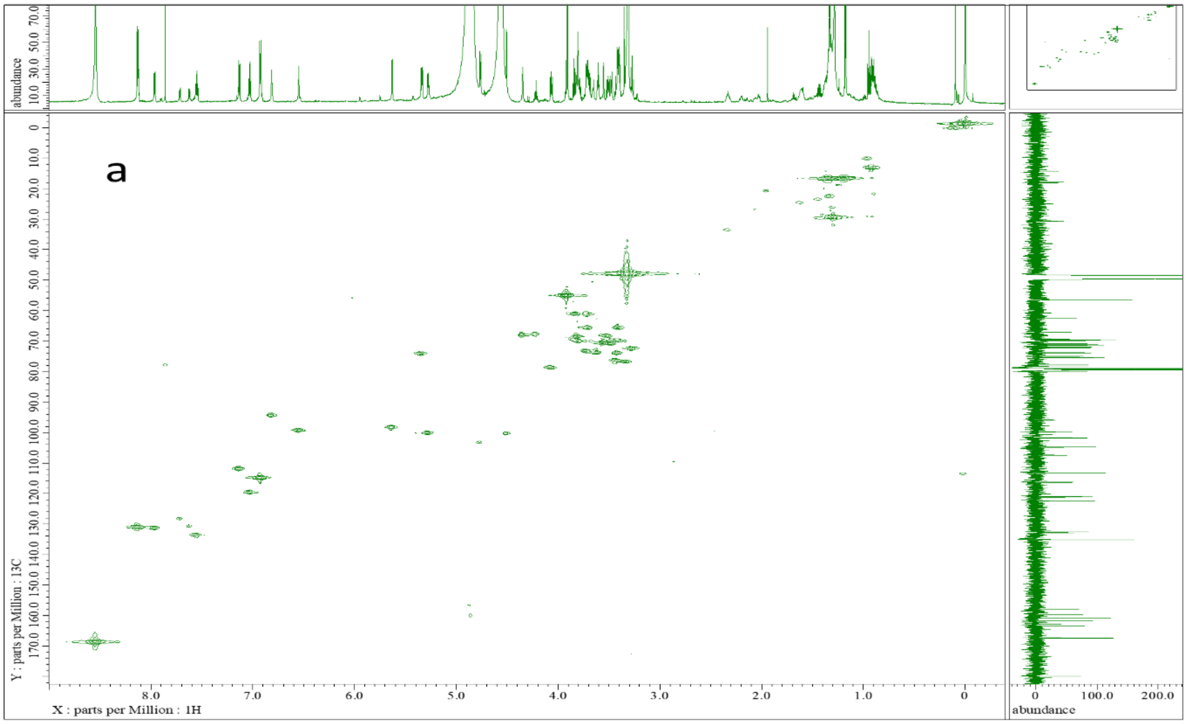

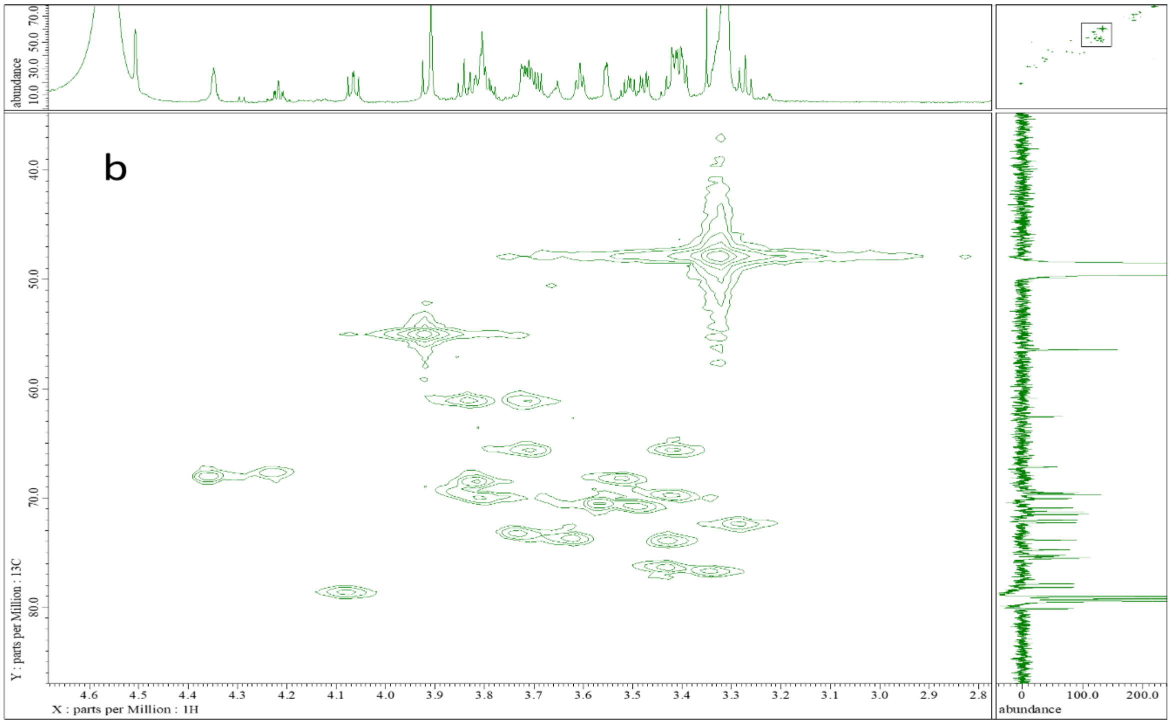

Figure S14. HMQC of compound **3** (CD_3OD, 800 MHz)

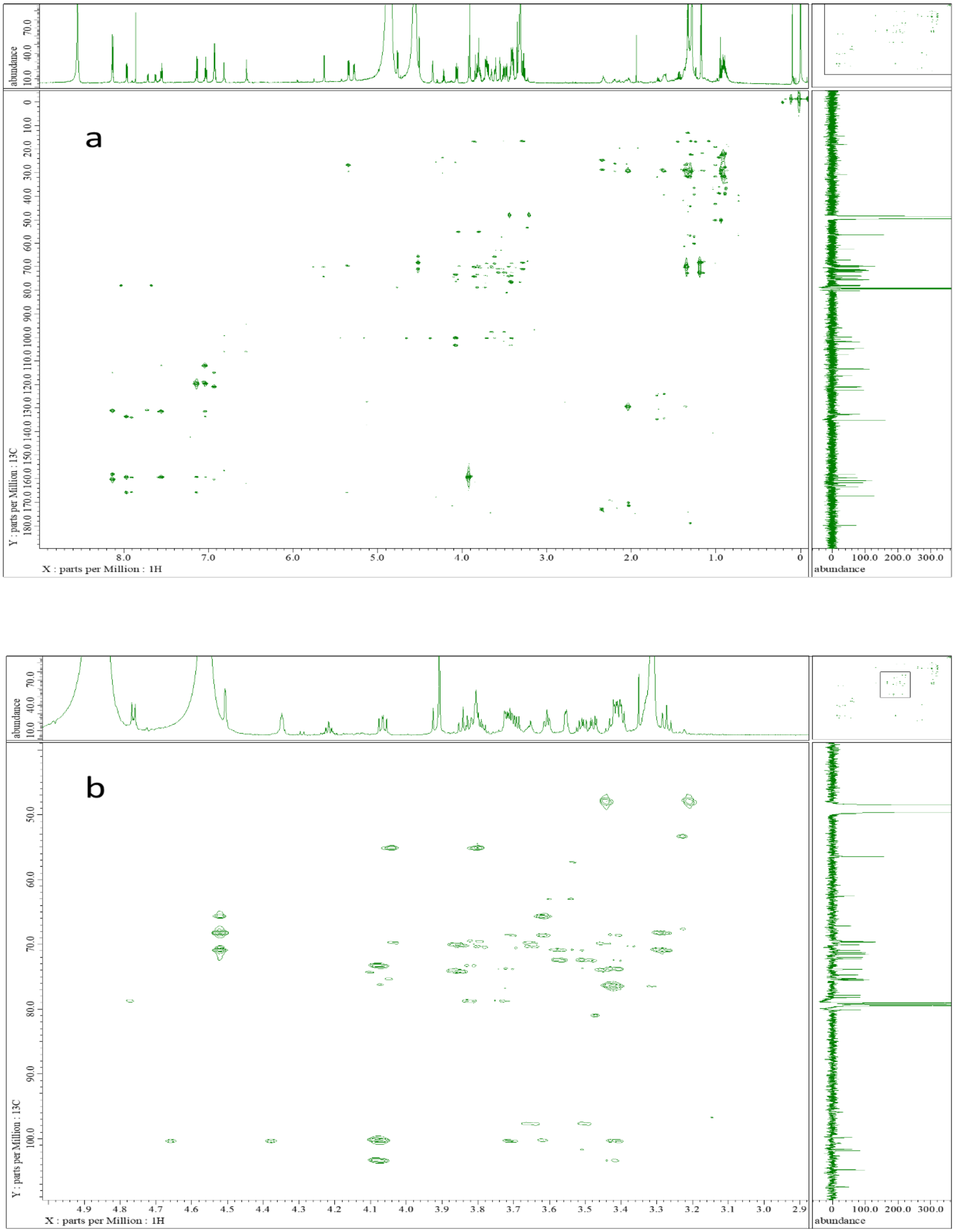

Figure S15. HMBC of compound **3** (CD_3OD, 800 MHz)

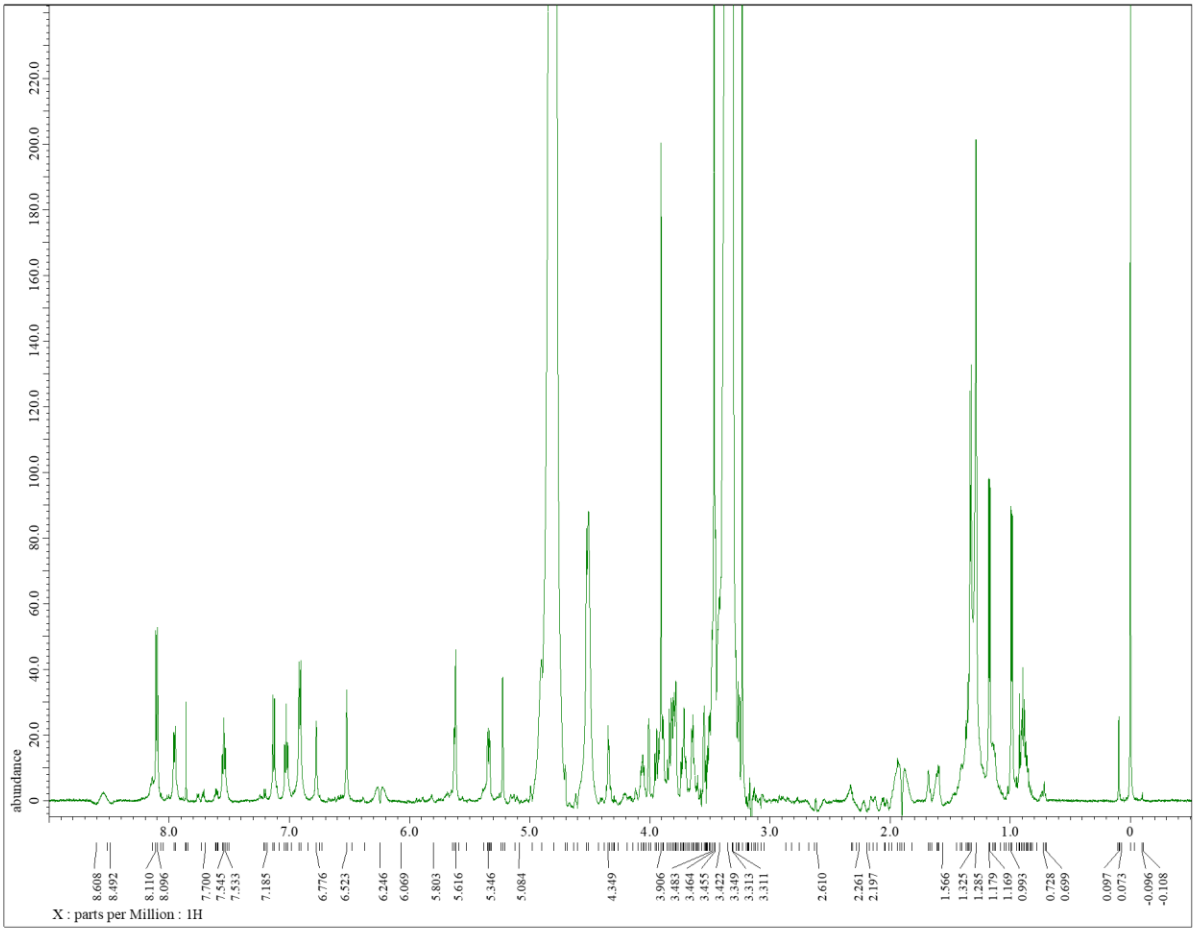

Figure S16. ^{1}H NMR spectrum of compound **4** (CD_3OD, 600 MHz)

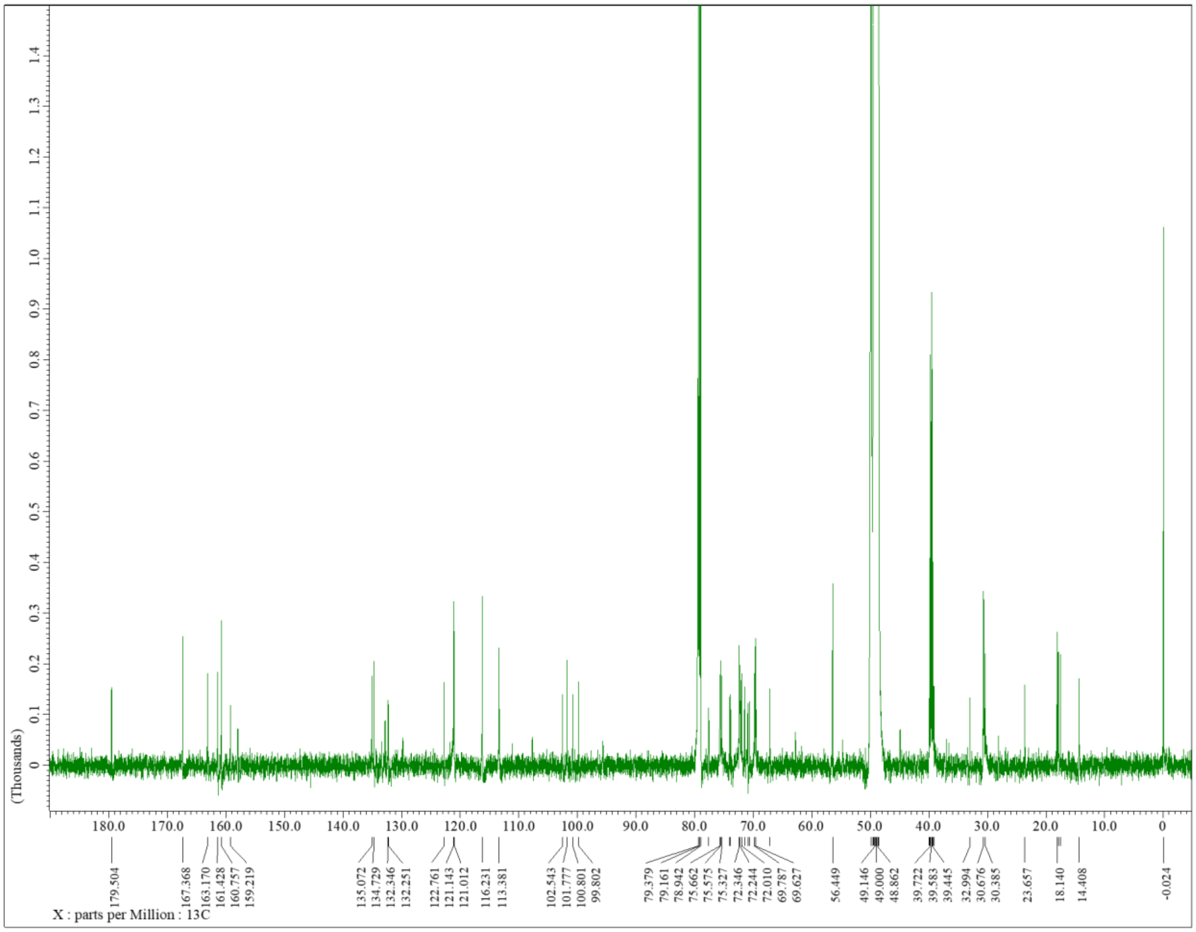

Figure S17. ^{13}C NMR spectrum of compound **4** (CD_3OD, 600 MHz)

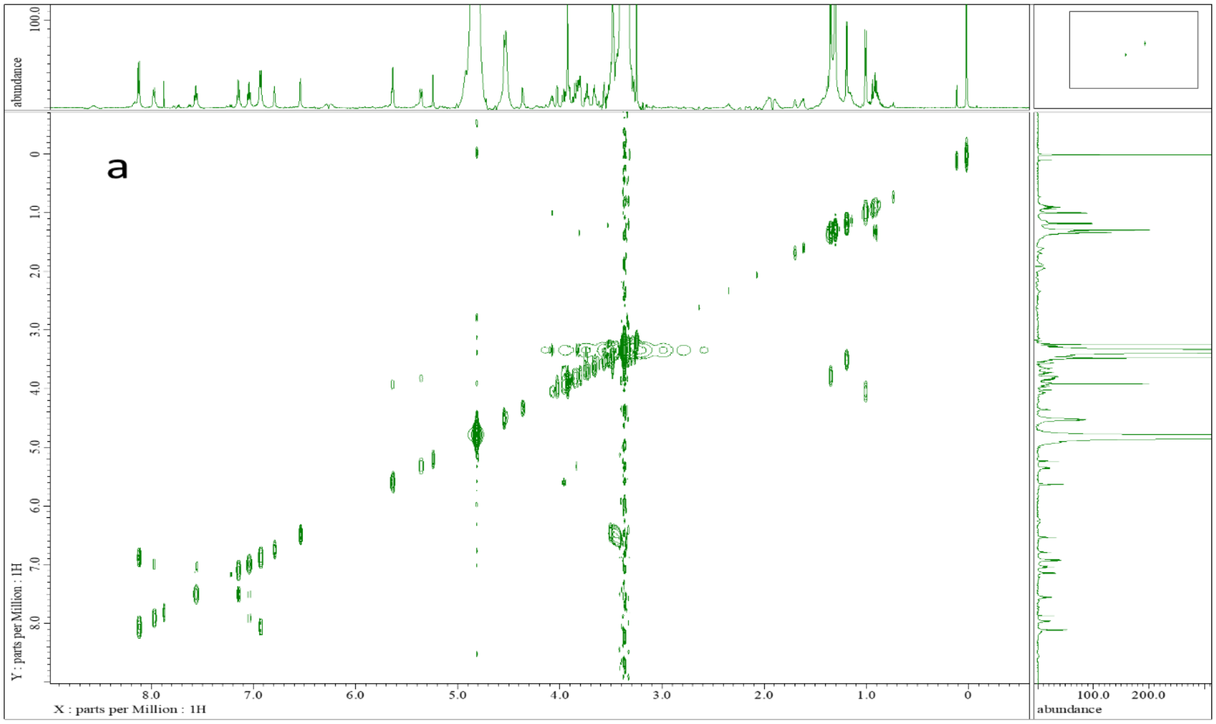

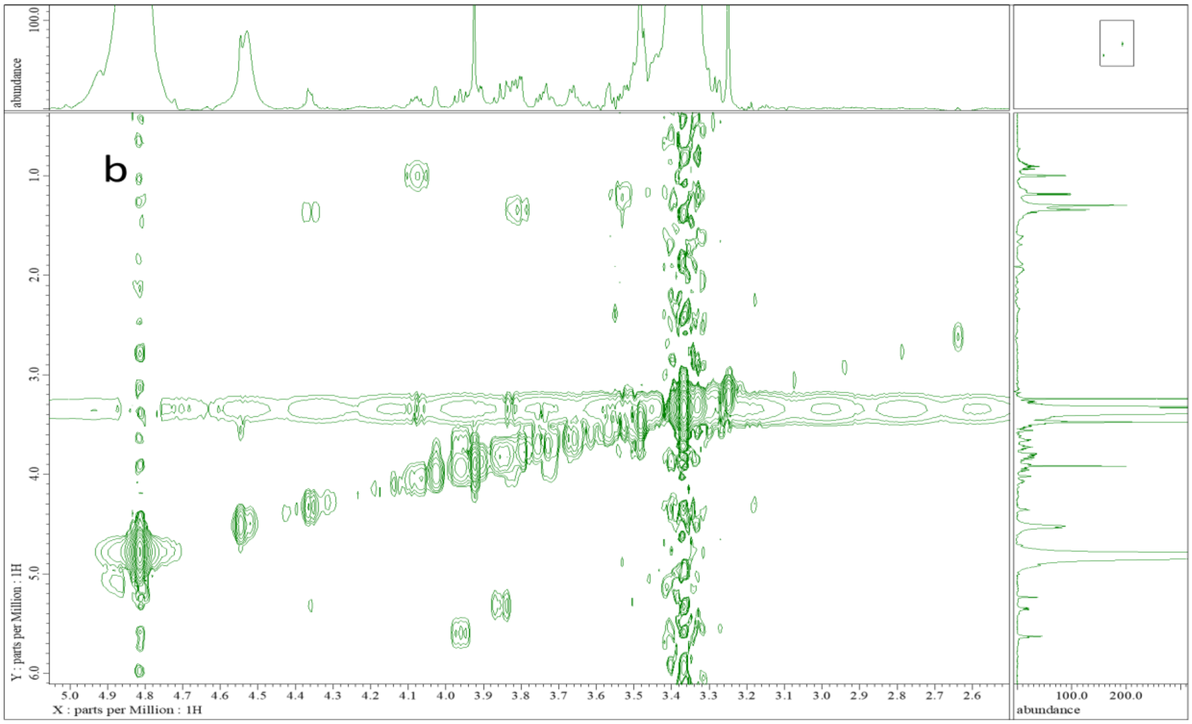

Figure S18. ^{1}H-^{1}H COSY of compound **4** (CD_3OD, 600 MHz)

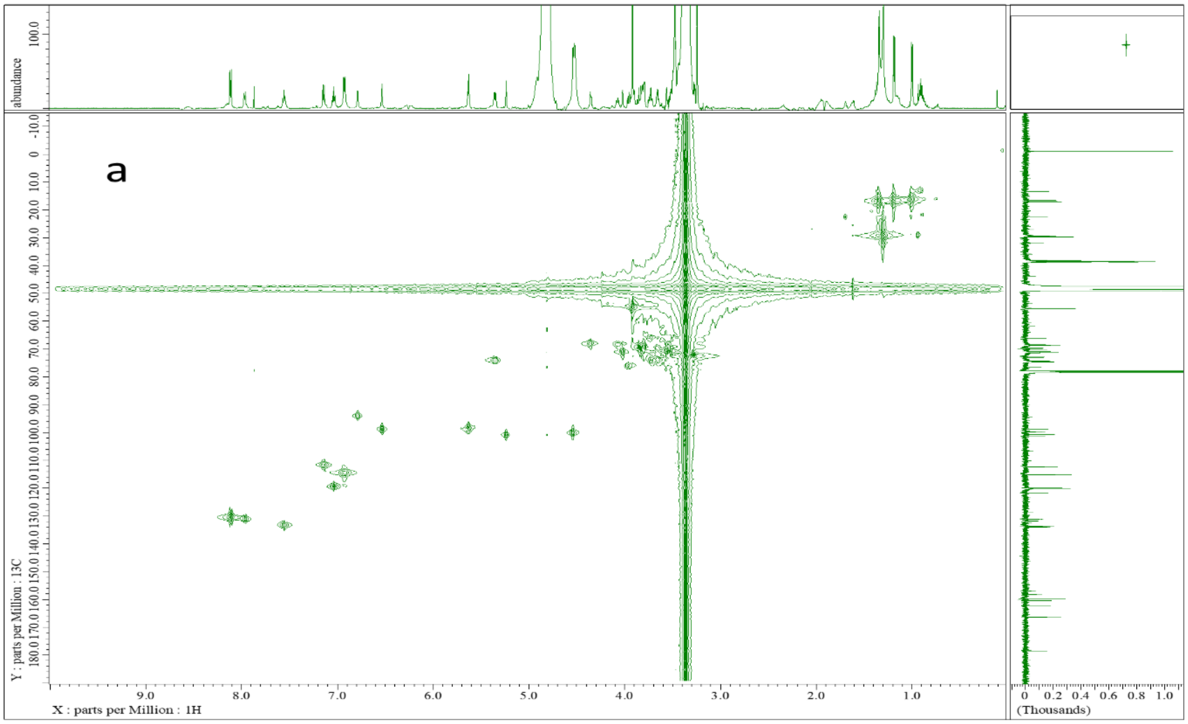

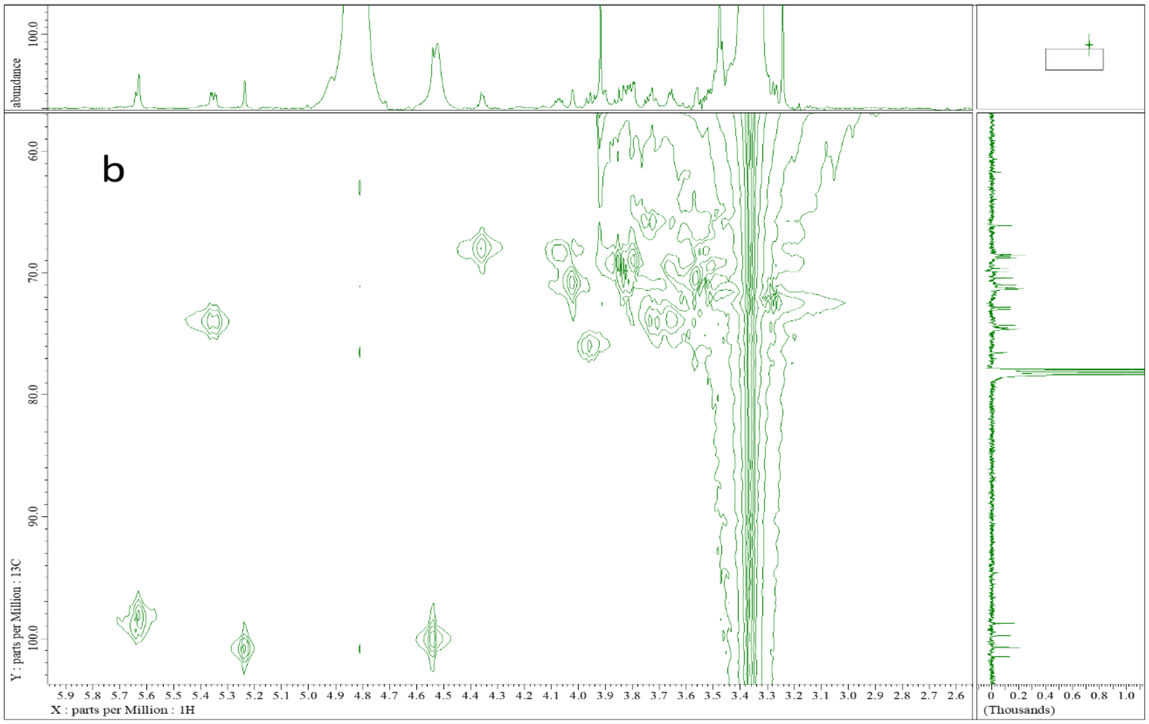

Figure S19. HMQC of compound **4** (CD_3OD, 600 MHz)

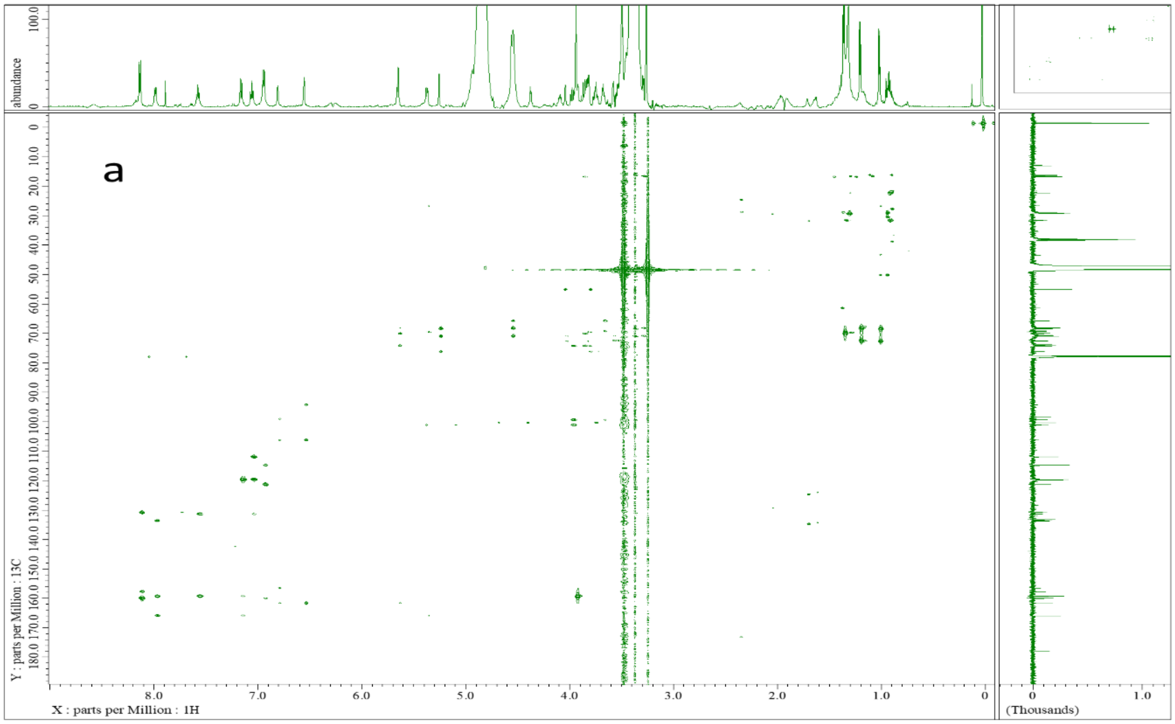

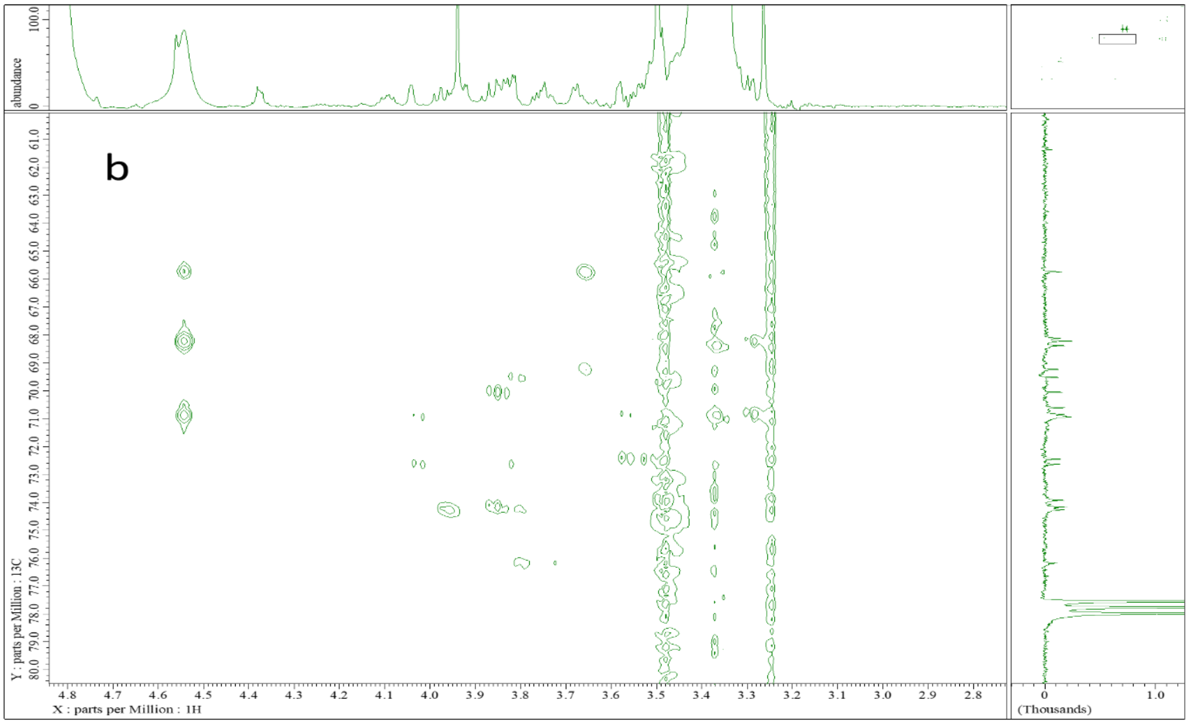

Figure S20. HMBC of compound **4** (CD_3OD, 600 MHz)

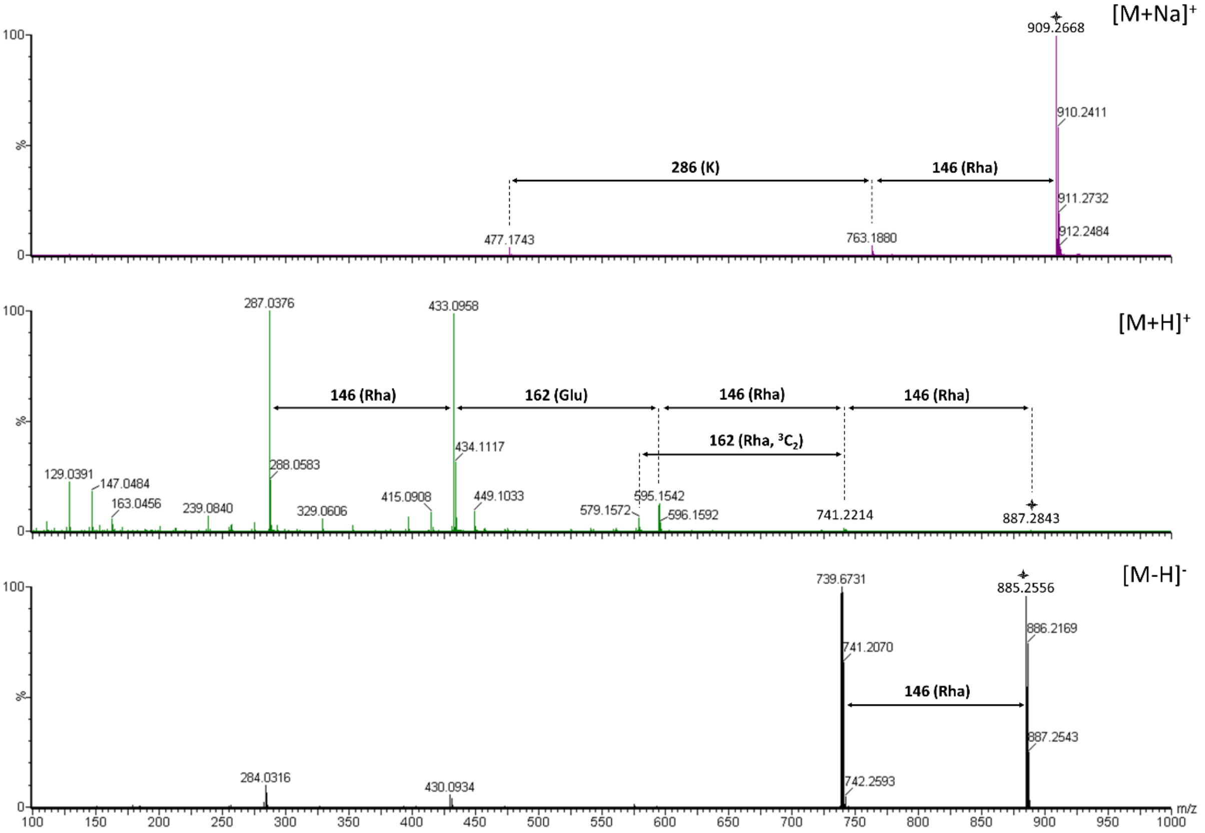

Figure S21. LC/ESI/MS/MS data of compound **1**

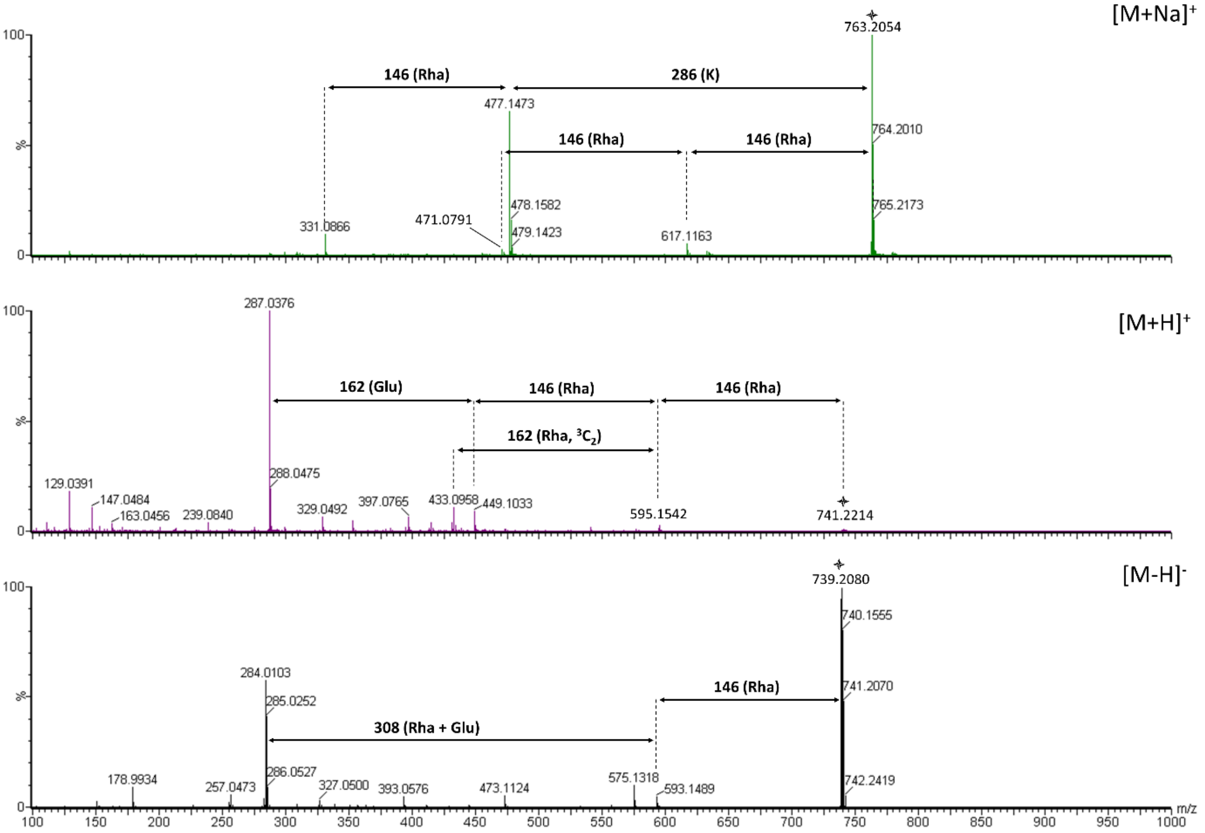

Figure S22. LC/ESI/MS/MS data of compound **2**

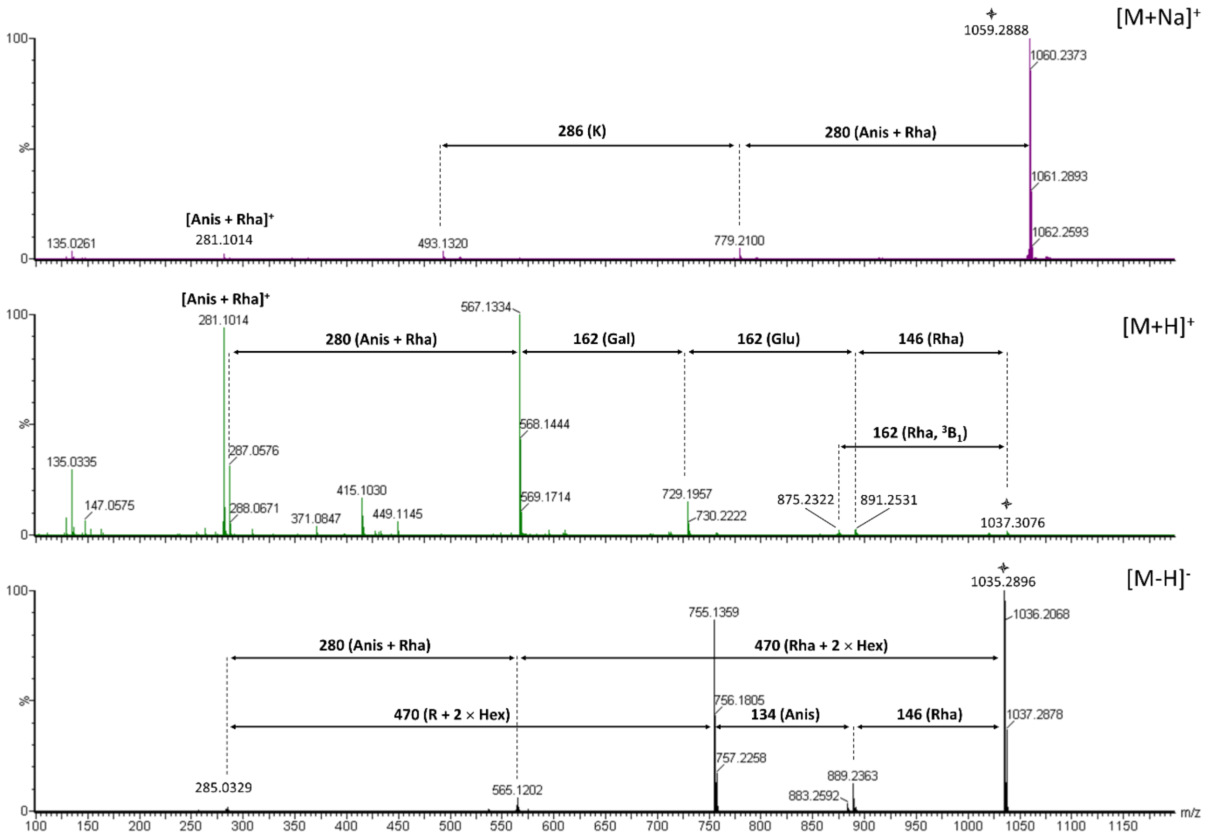

Figure S23. LC/ESI/MS/MS data of compound **3**

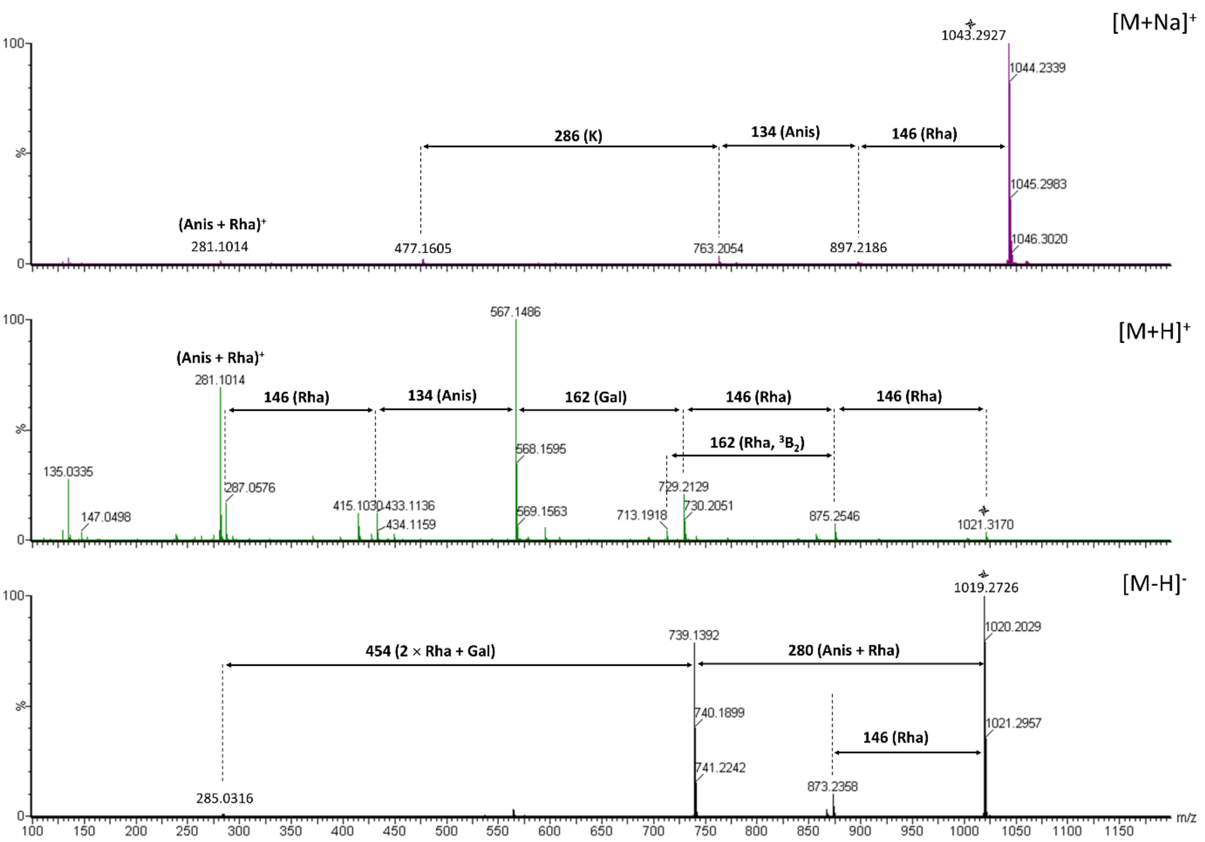

Figure S24. LC/ESI/MS/MS data of compound **4**

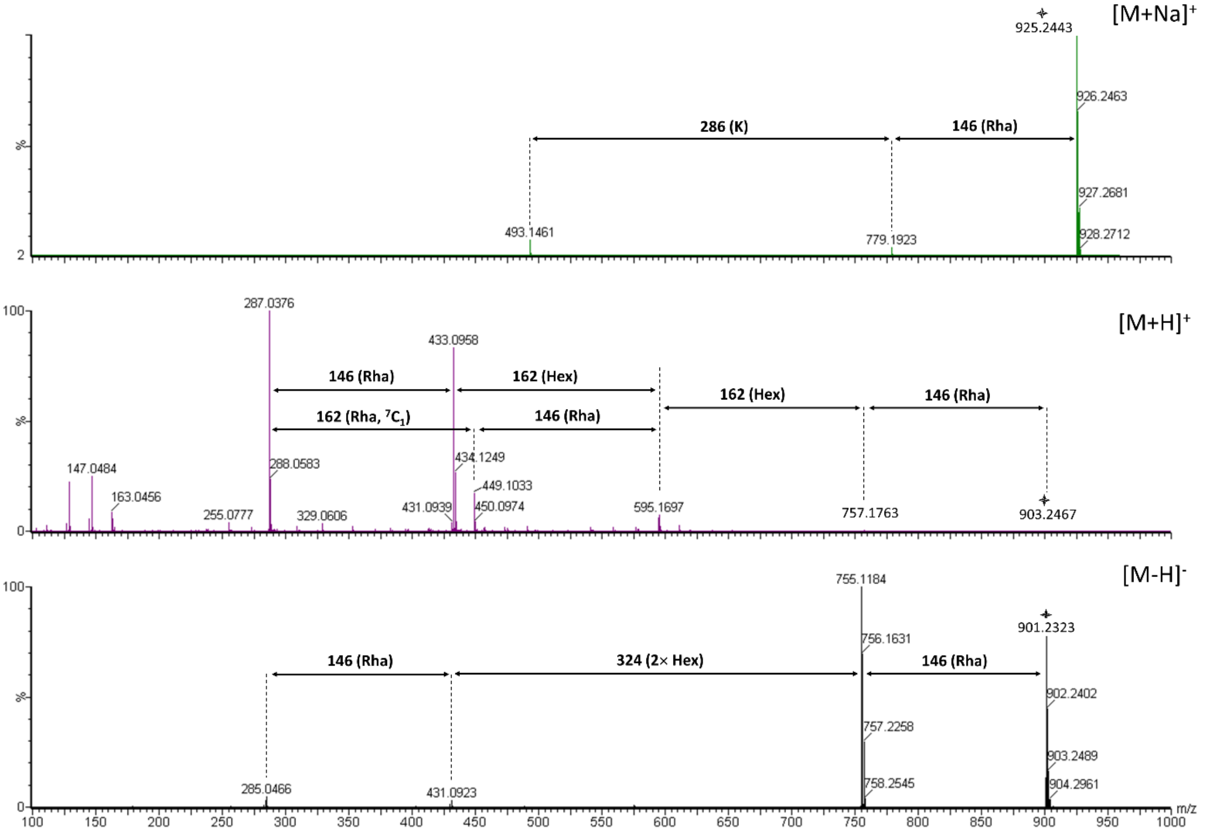

Figure S25. LC/ESI/MS/MS data of compound **5**

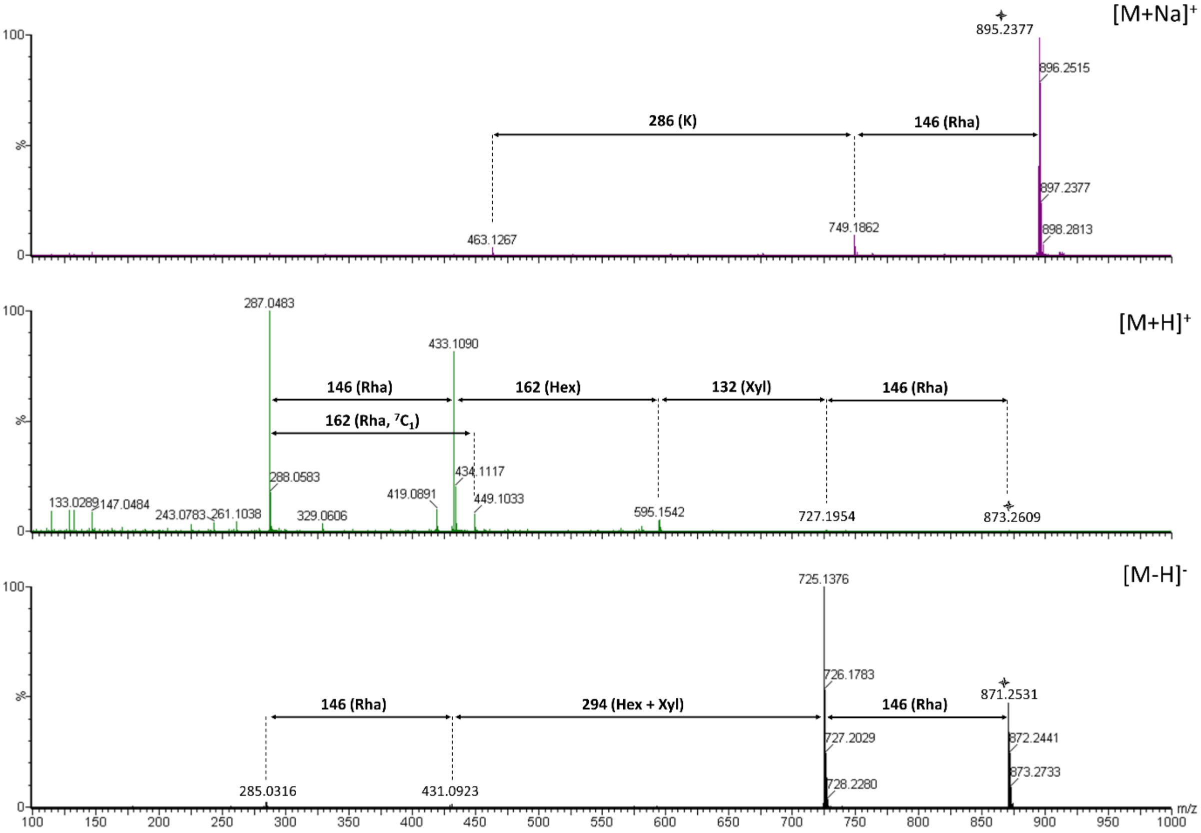

Figure S26. LC/ESI/MS/MS data of compound **6**

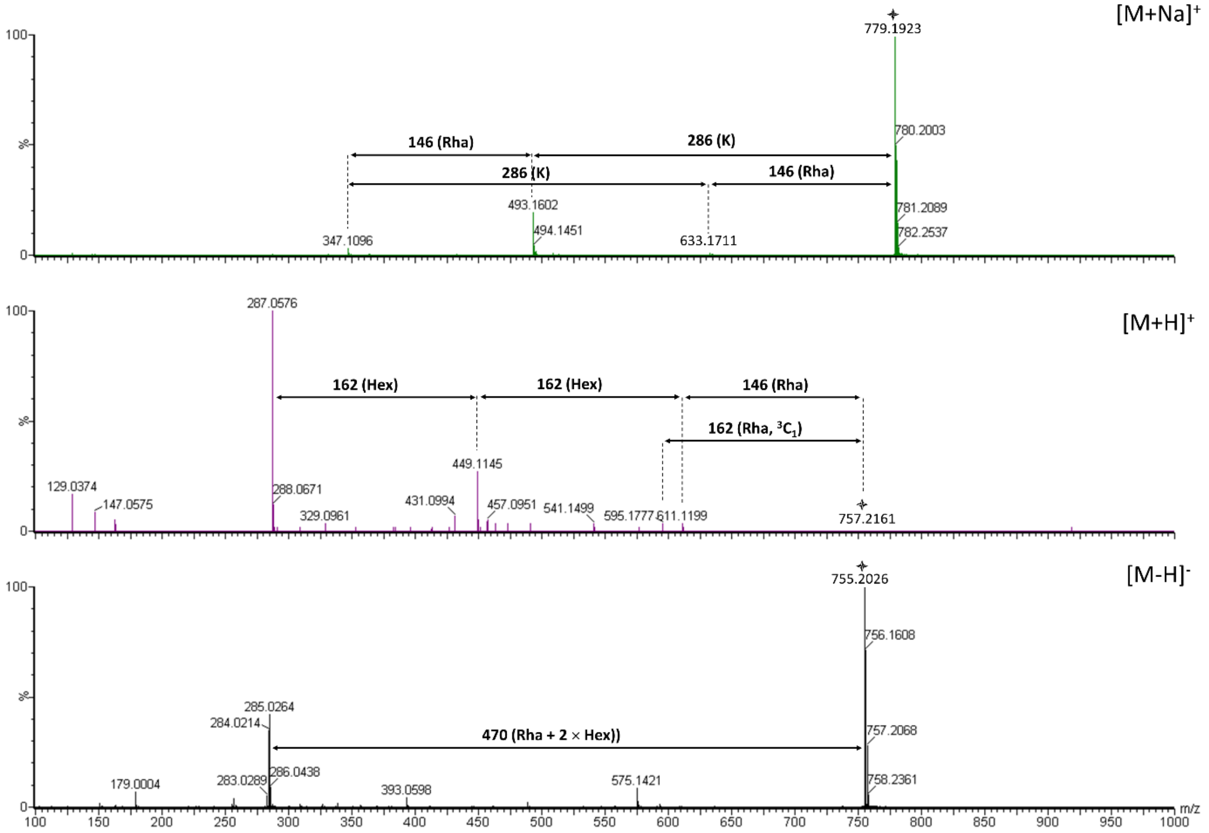

Figure S27. LC/ESI/MS/MS data of compound **7**

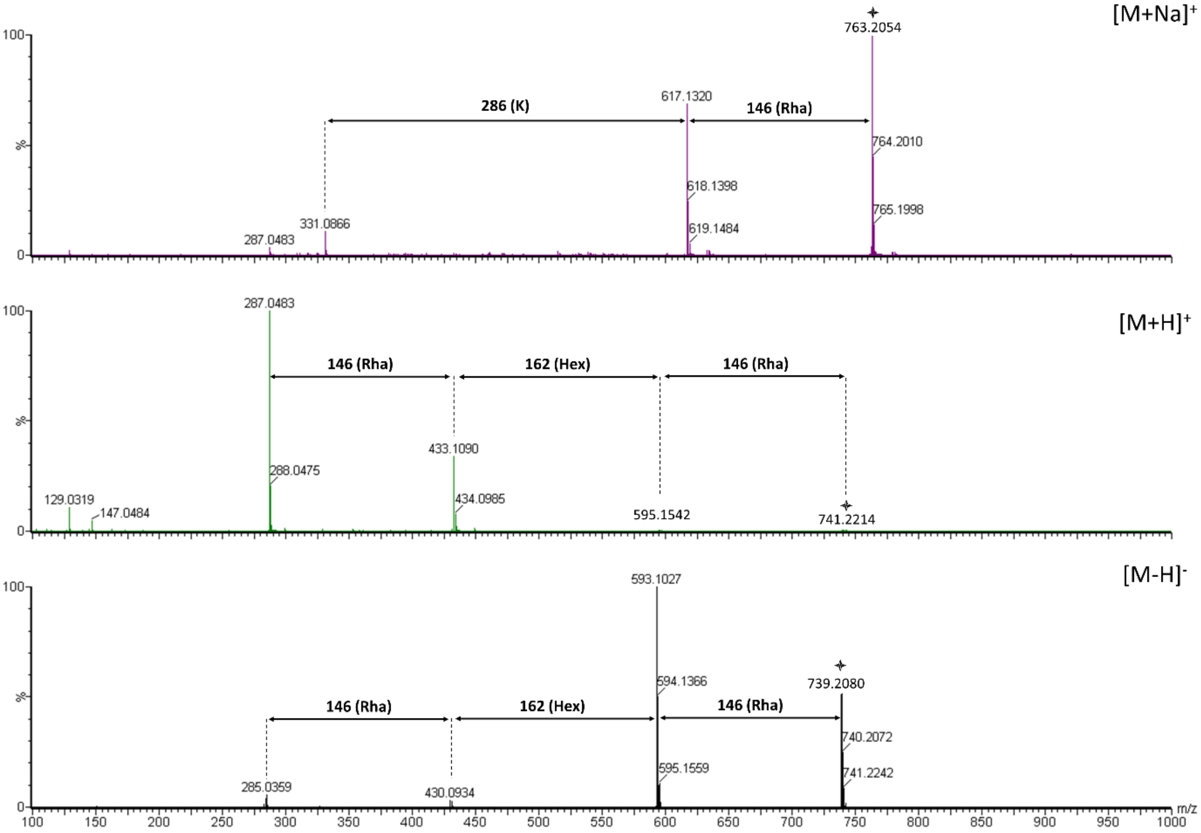

Figure S28. LC/ESI/MS/MS data of compound **8**

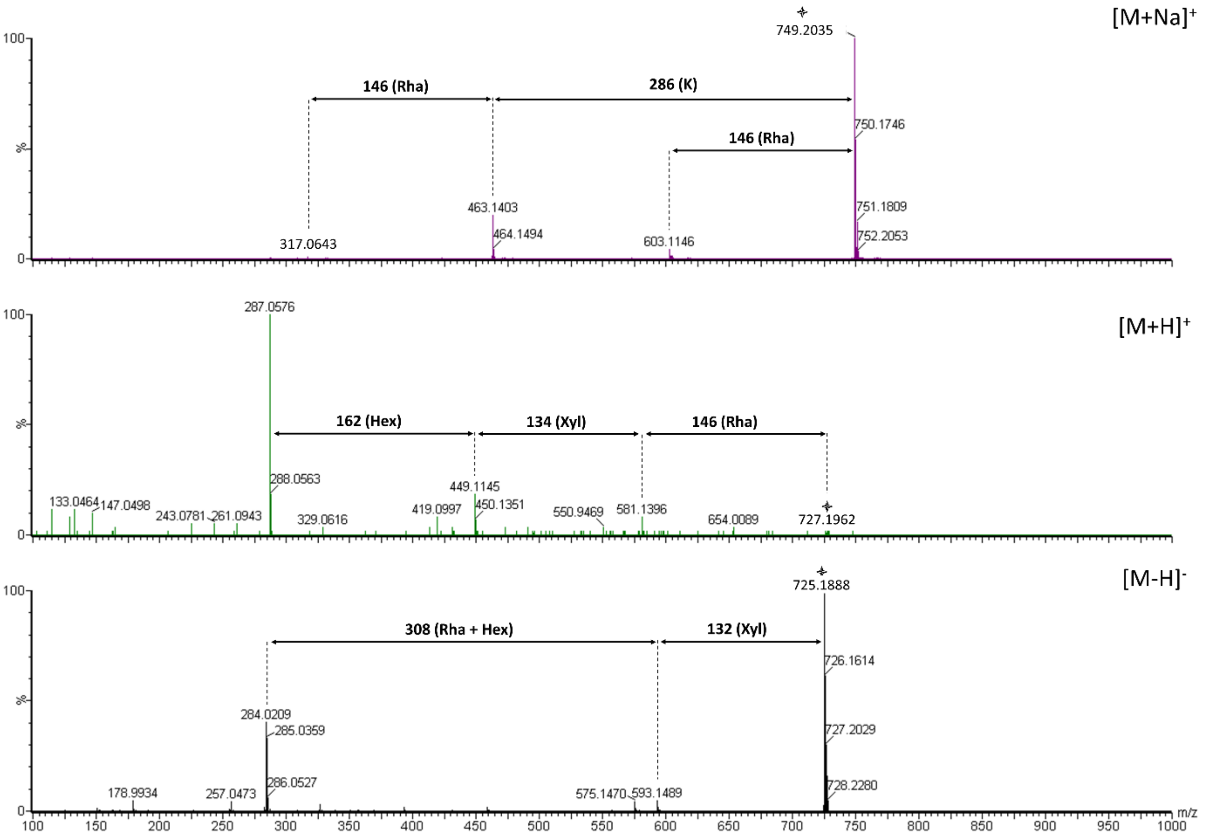

Figure S29. LC/ESI/MS/MS data of compound **9**

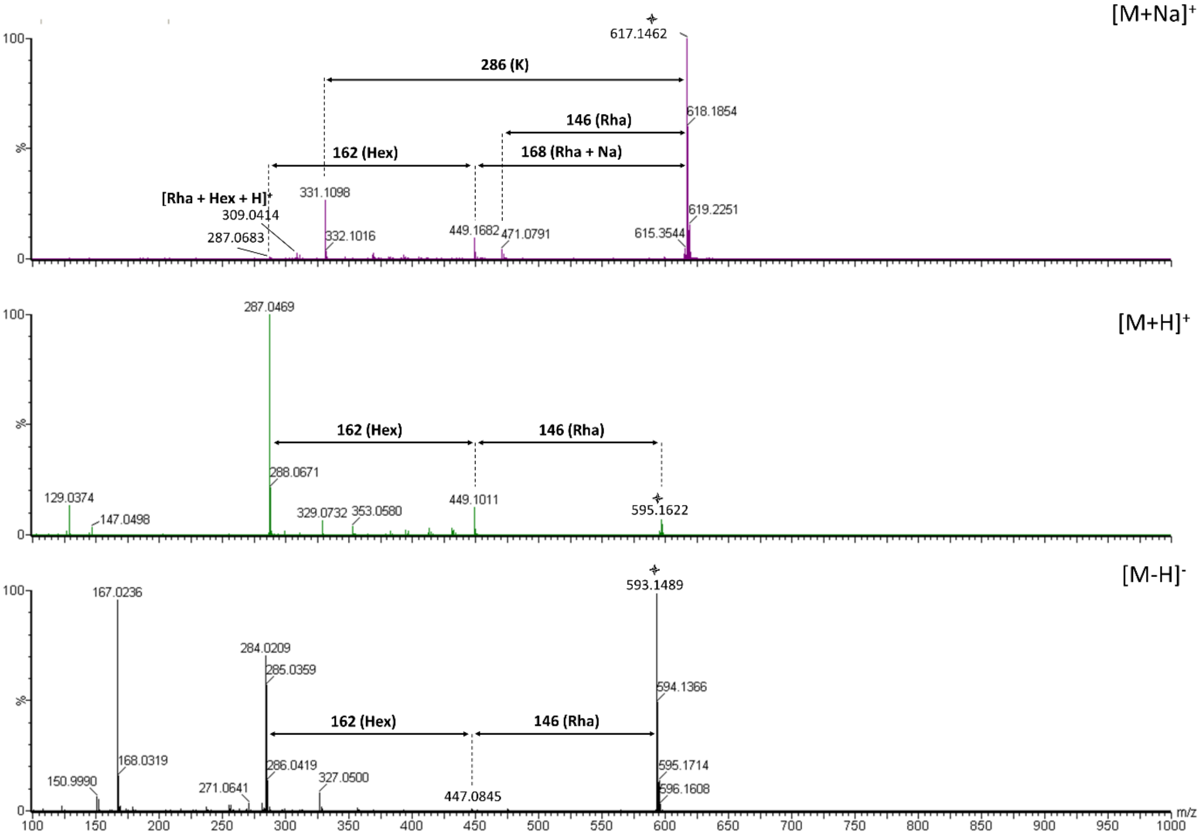

Figure S30. LC/ESI/MS/MS data of compound **10**

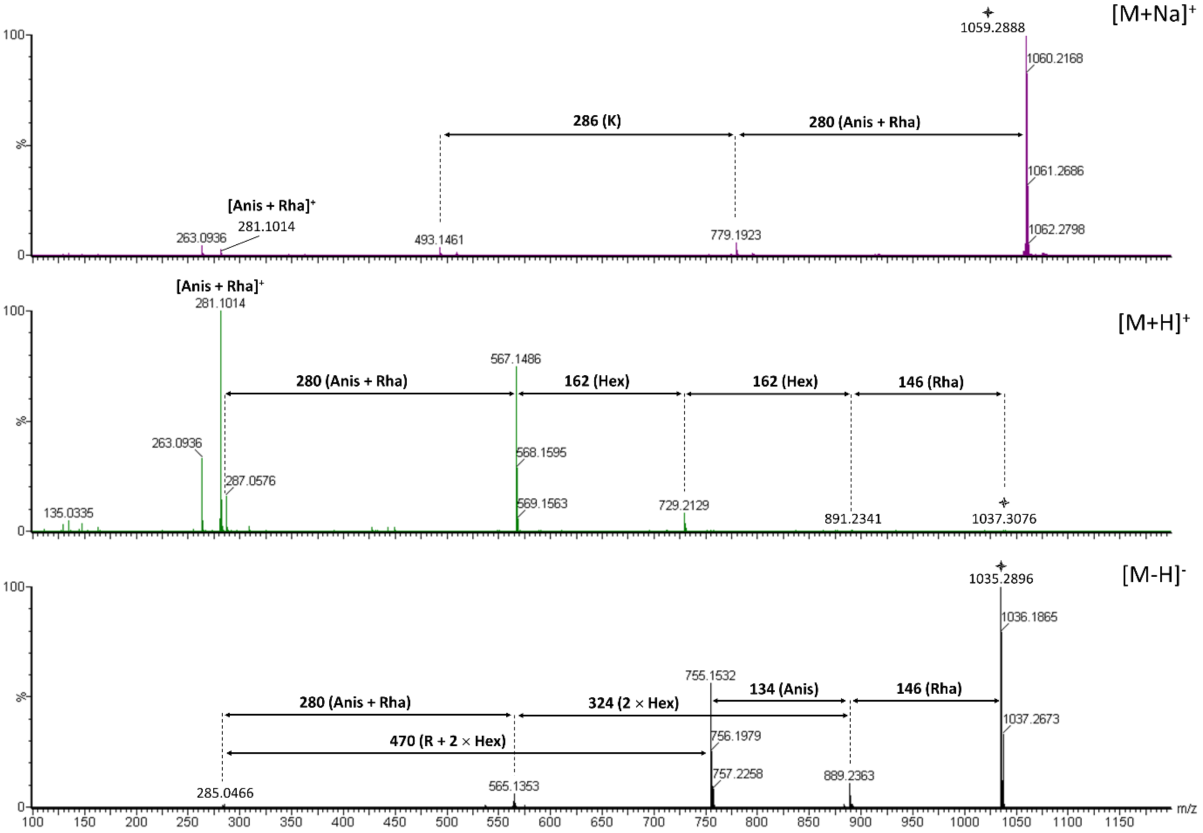

Figure S31. LC/ESI/MS/MS data of compound **11**

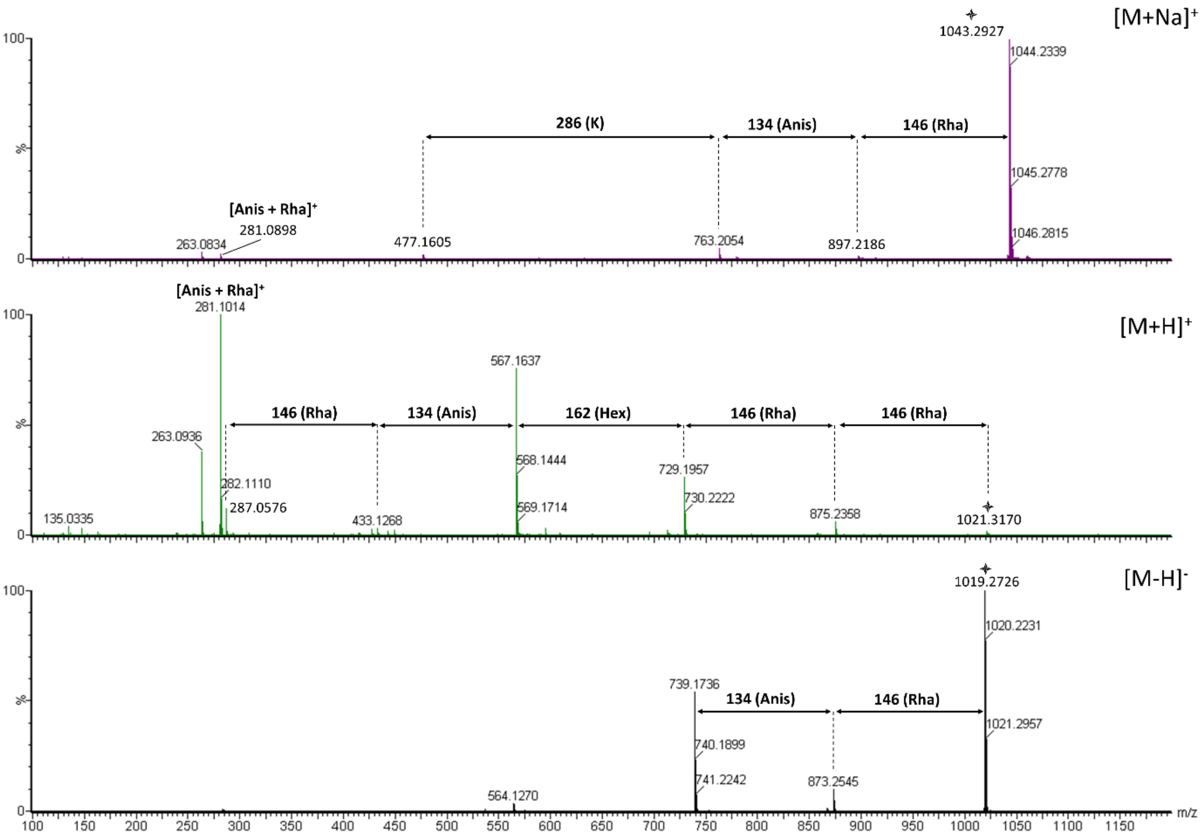

Figure S32. LC/ESI/MS/MS data of compound **12**

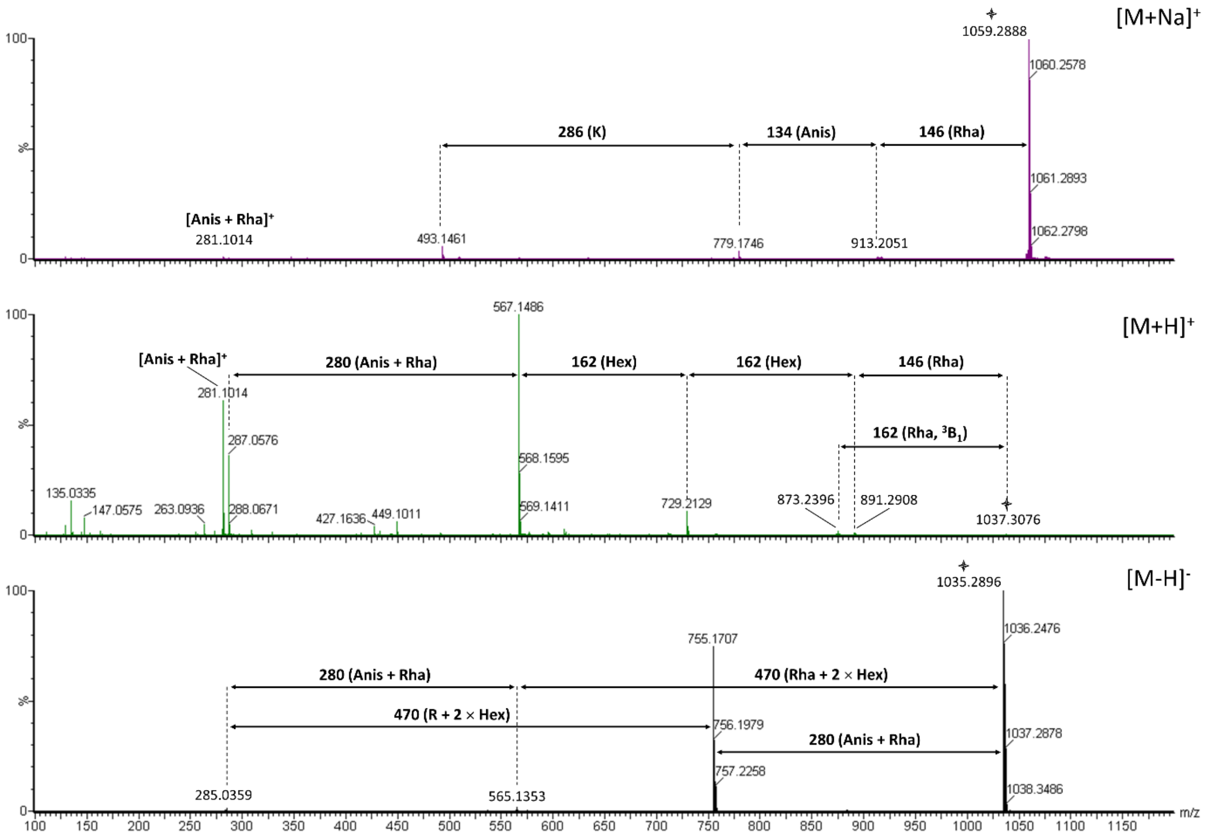

Figure S33. LC/ESI/MS/MS data of compound **13**

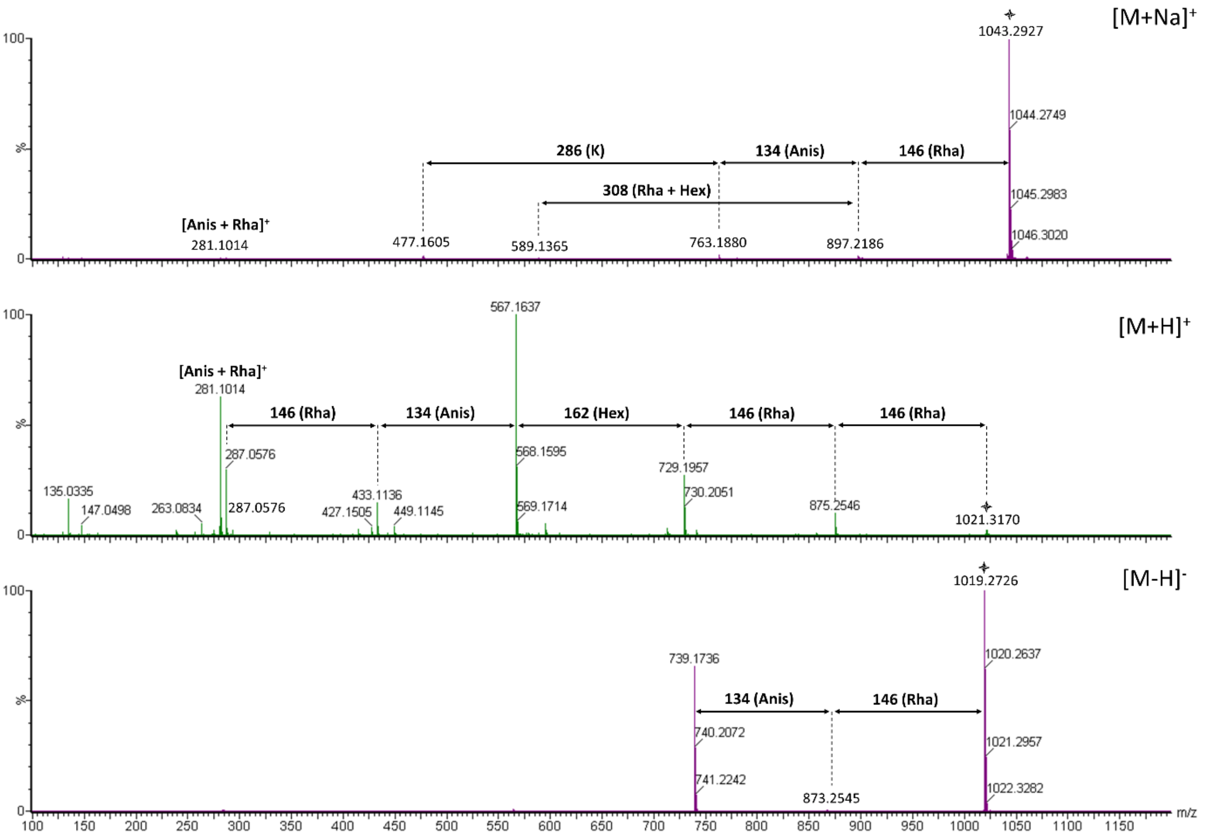

Figure S34. LC/ESI/MS/MS data of compound **14**

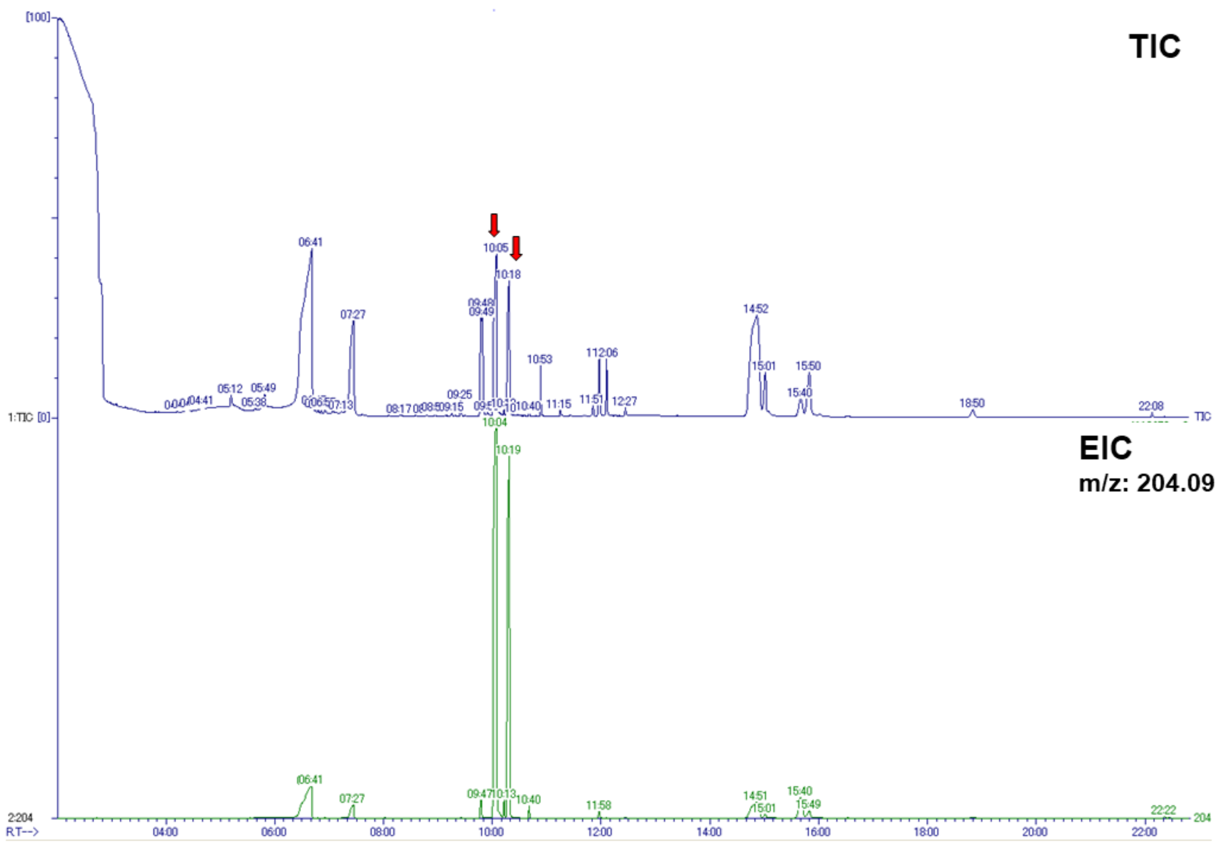

Figure S35. GC-MS total ion chromatogram and extracted ion chromatogram of trimethylsilyl (TMS) derivatives of galactose

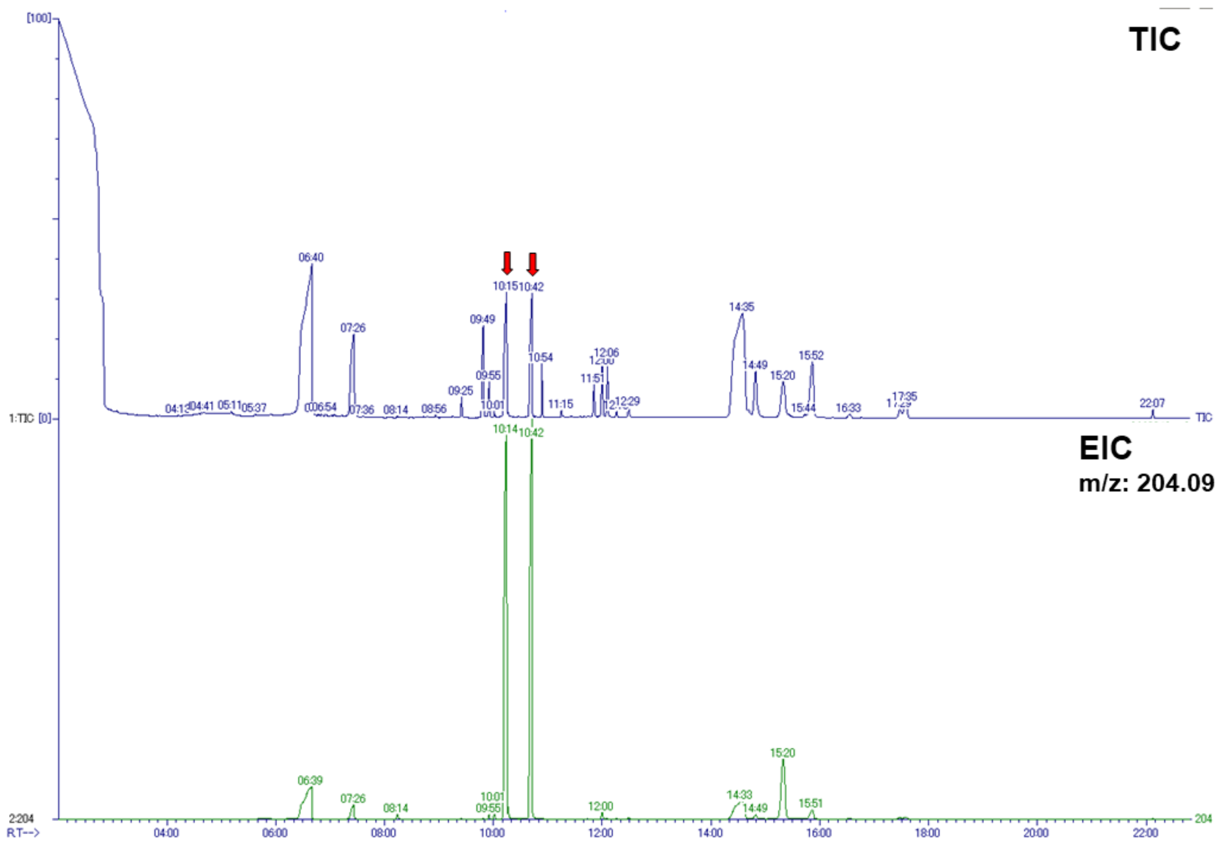

Figure S36. GC-MS total ion chromatogram and extracted ion chromatogram of trimethylsilyl (TMS) derivatives of glucose

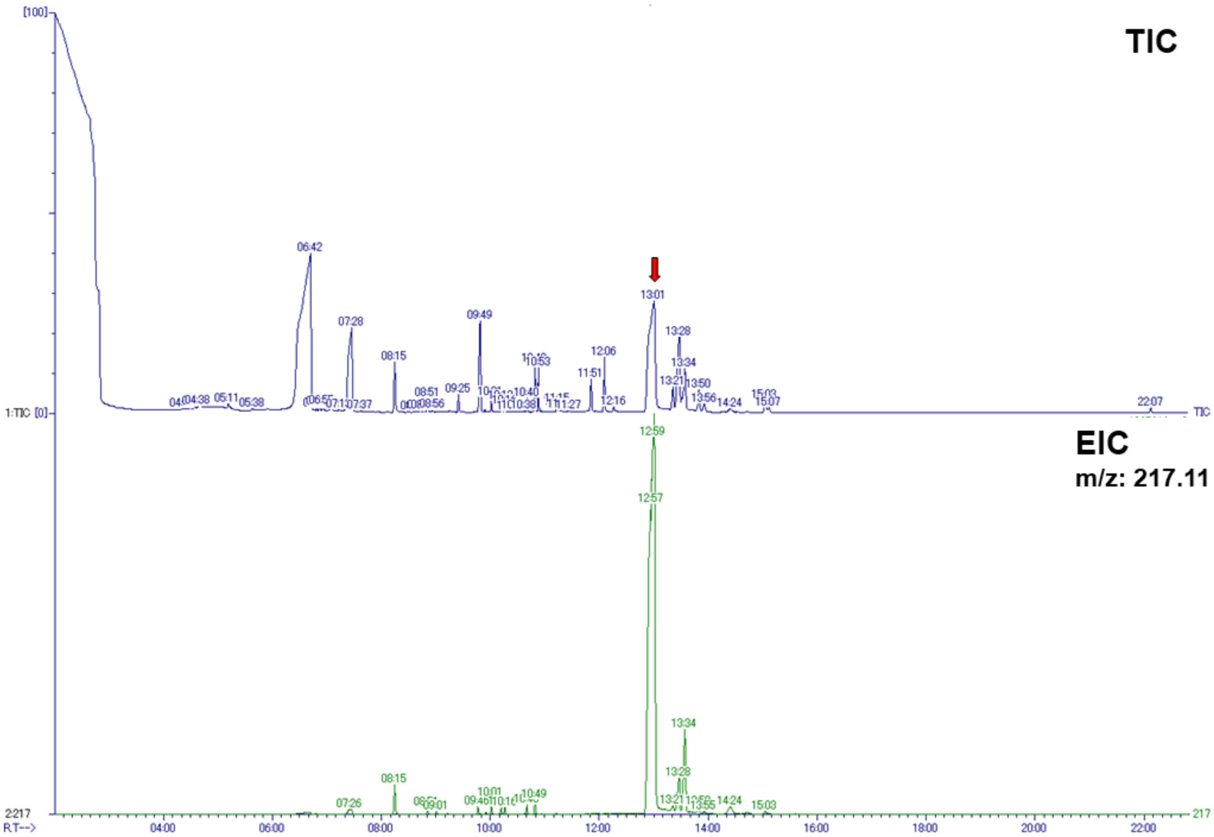

Figure S37. GC-MS total ion chromatogram and extracted ion chromatogram of trimethylsilyl (TMS) derivatives of rhamnose

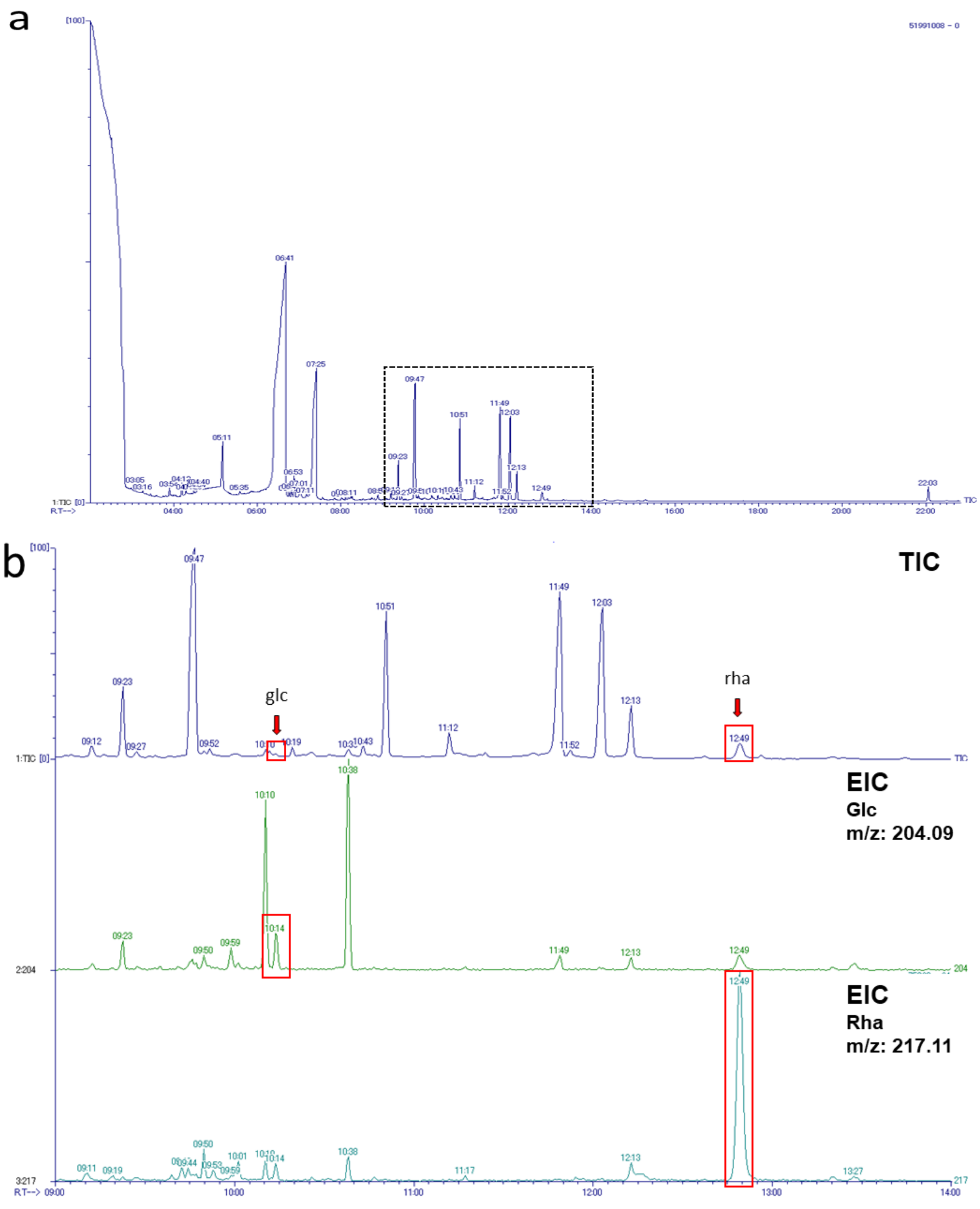

Figure S38. GC-MS total ion chromatogram and extracted ion chromatogram trimethylsilyl (TMS) derivatives of compound **1**. (a) full retention time chromatogram, (b) expand on retention time chromatogram.

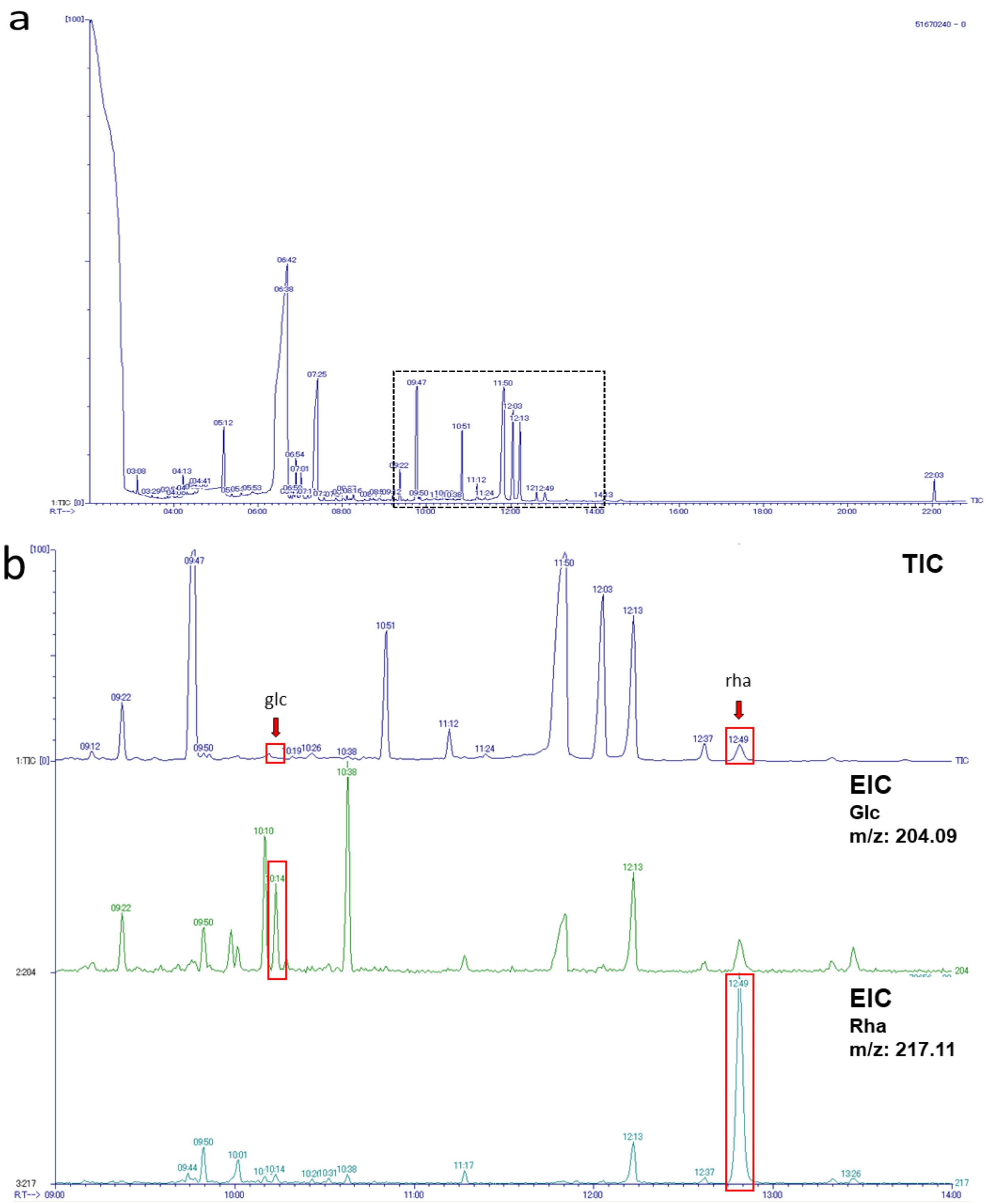

Figure S39. GC-MS total ion chromatogram and extracted ion chromatogram trimethylsilyl (TMS) derivatives of compound **2**. (a) full retention time chromatogram, (b) expand on retention time chromatogram.

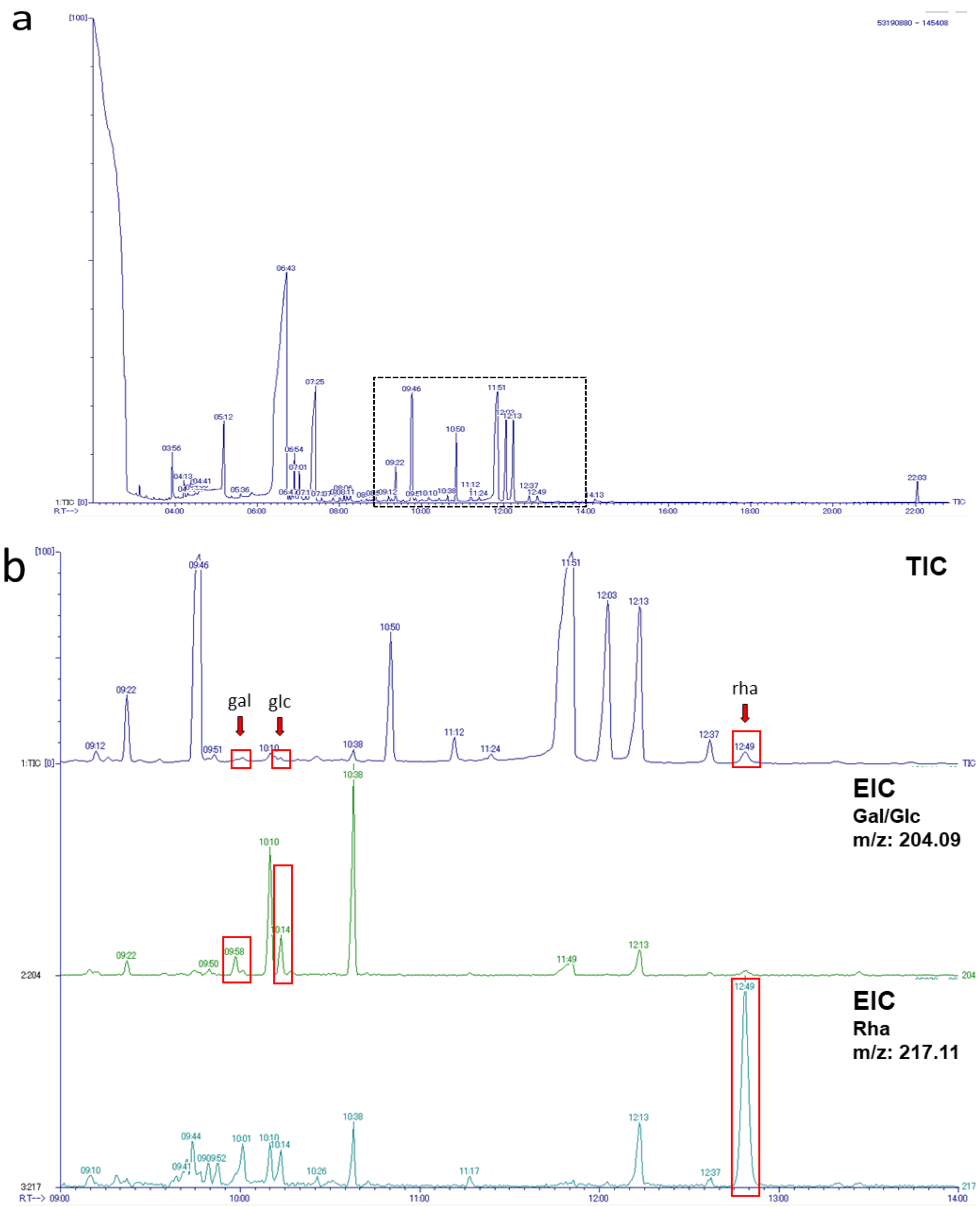

Figure S40. GC-MS total ion chromatogram and extracted ion chromatogram trimethylsilyl (TMS) derivatives of compound **3**. (a) full retention time chromatogram, (b) expand on retention time chromatogram.

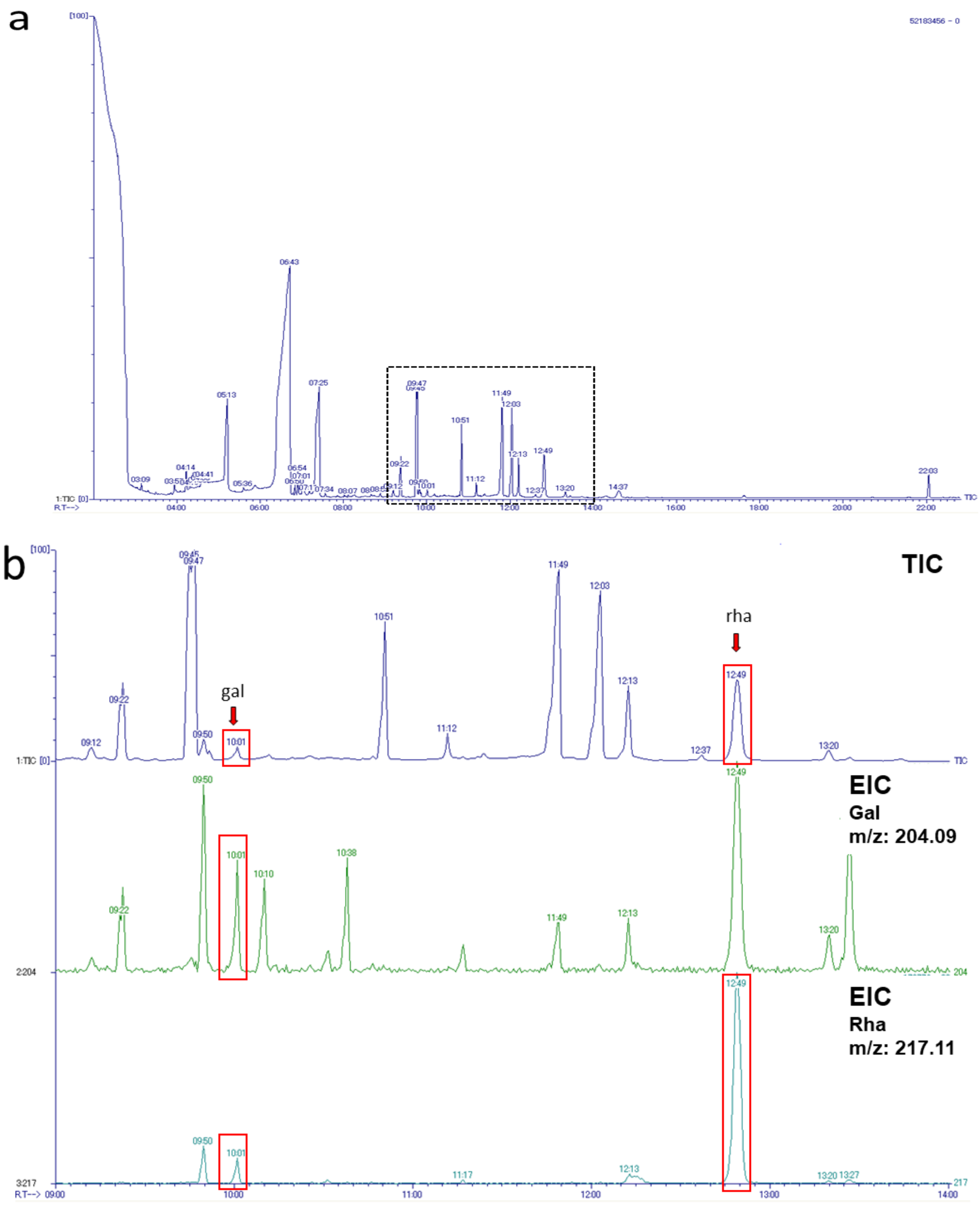

Figure S41. GC-MS total ion chromatogram and extracted ion chromatogram trimethylsilyl (TMS) derivatives of compound **4**. (a) full retention time chromatogram, (b) expand on retention time chromatogram.

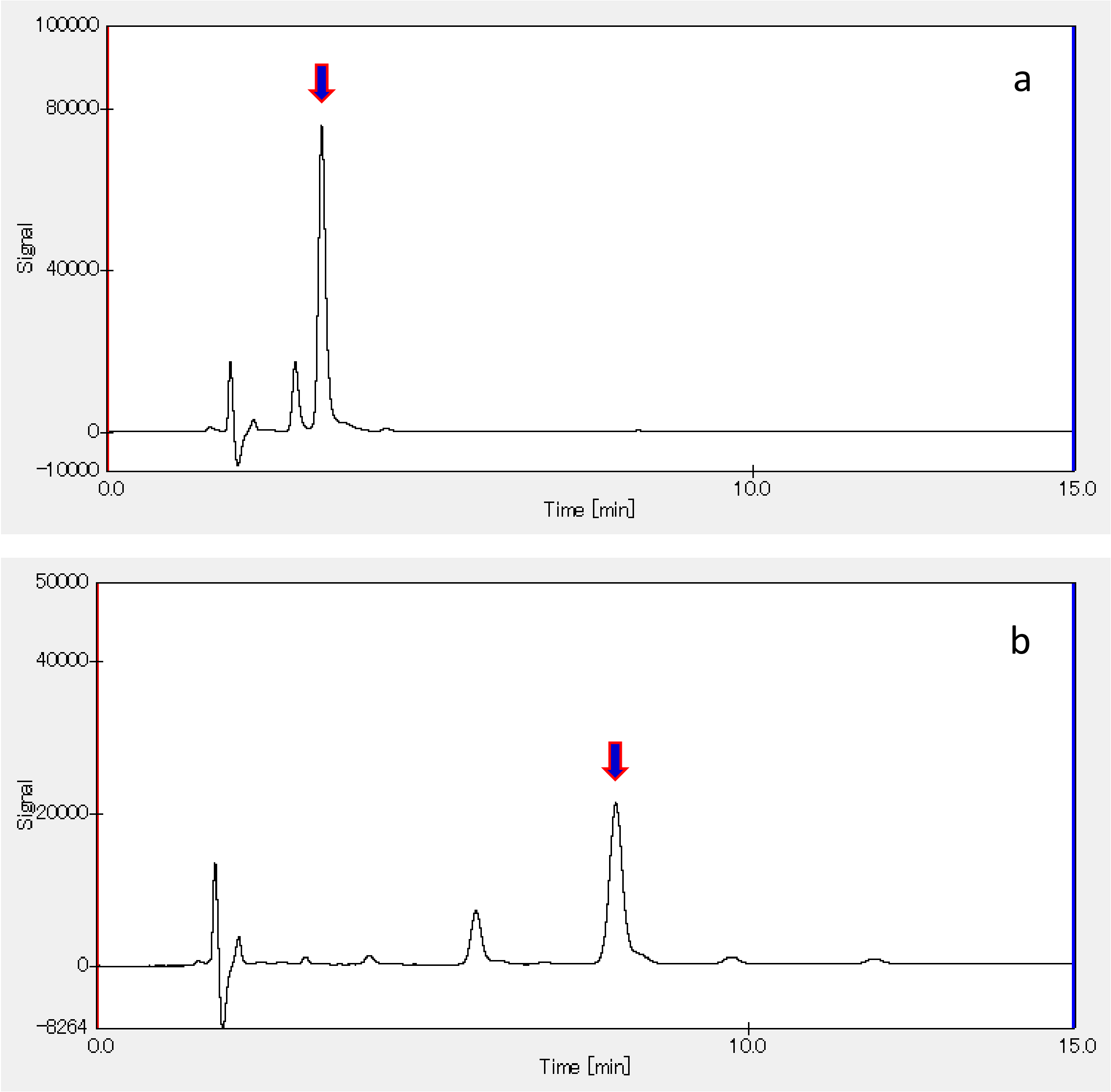

Figure S42. Chromatogram of compounds **1** (a) and **2** (b)

Note:

HPLC was performed using a Jasco PU2089 intelligent pump equipped with a Jasco UV-2075 detector (Tokyo, Japan) and a Shimadzu CTO-10ACVP column oven (Kyoto, Japan). Cosmosil 5C18-MS-II column (4.6 mm I.D. × 150 mm, particle size of 5 µm) was used and the temperature was 35 °C. Elution was performed with 15% MeCN and 0.5% HCOOH in water. The flow rate of the eluent was 1.0 mL/min.

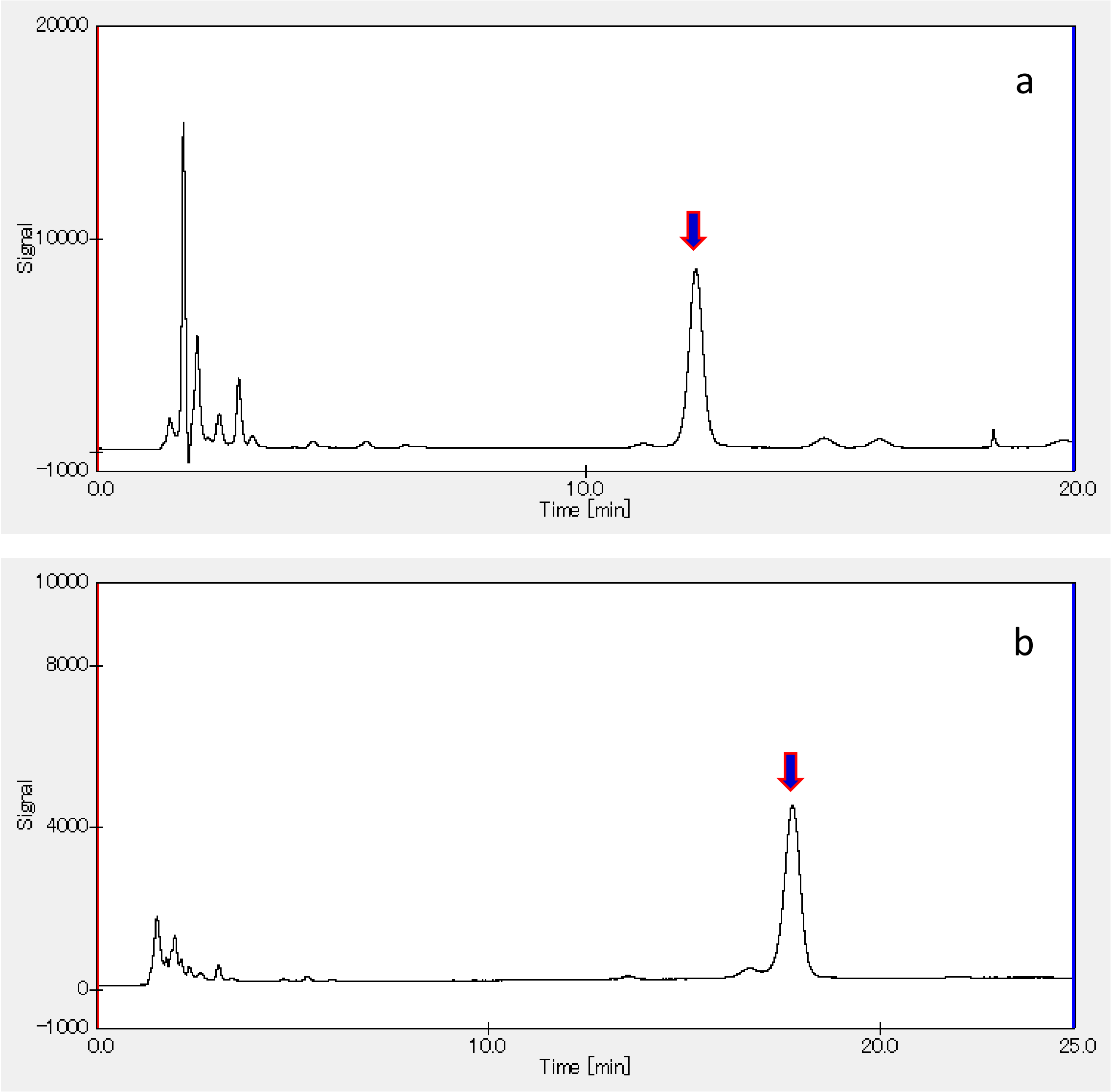

Figure S43. Chromatogram of compounds **3** (a) and **4** (b)

Note:

HPLC was performed using a Jasco PU2089 intelligent pump equipped with a Jasco UV-2075 detector (Tokyo, Japan) and a Shimadzu CTO-10ACVP column oven (Kyoto, Japan). Cosmosil 5C18-MS-II column (4.6 mm I.D. × 150 mm, particle size of 5 µm) was used and the temperature was 35 °C. Elution was performed with 20 % MeCN and 0.5% HCOOH in water. The flow rate of the eluent was 1.0 mL/min.
